# Comparison of Xanthine Oxidase Inhibitory Activities and Phenolic, Fatty Acid, Element, and Vitamin Levels of Four Mushroom Species

**DOI:** 10.1002/fsn3.70203

**Published:** 2025-04-21

**Authors:** Suat Ekin, Mahire Bayramoglu Akkoyun, Ahmet Bakir, Mustafa Emre Akcay, Emre Can Ekin

**Affiliations:** ^1^ Department of Chemistry, Science Faculty Van Yuzuncu Yil University Van Turkiye; ^2^ Division of Biochemistry, Faculty of Veterinary Medicine Siirt University Siirt Turkiye; ^3^ Department Biology, Faculty of Science Yuzuncu Yil University Van Turkiye; ^4^ Nursing Department, Faculty of Health Sciences Istanbul Arel University Istanbul Turkiye

**Keywords:** edible mushrooms, fatty acid, phenolic compound, trace element, vitamin, xanthine oxidase

## Abstract

The purpose of this study was to determine the antioxidant properties and the composition of phenolic compounds, trace elements, vitamins, and fatty acids in the edible mushrooms *Helvella leucopus, Tricholoma terreum, Lepista nuda,* and *Marasmius oreades*, as well as their inhibitory effect on xanthine oxidase. Elemental analyses were conducted using ICP‐OES. Phenolic compounds and vitamins were performed by HPLC, while identification of fatty acids was performed by GC–MS. HPLC analysis revealed the phenolic compounds in *H. leucopus, T. terreum, L. nuda, and M. oreades*, with gallic acid being the main compound identified, with levels of 133.04, 246.49, 408.64, and 129.302 μg/g dry weight, respectively. For vitamins, α‐tocopherol is the most primary vitamin found with values of 0.6009, 0.79, and 0.3581 μmol/kg dw. GC–MS analysis determined that the fatty acids with linoleic acid are the major fatty acids identified, with percentages of 30.82%, 19.92%, 30.38%, and 20.86%. ICP‐OES measurement indicated that trace elements with iron as the dominant trace element were observed, with concentrations of 0.248, 0.237, 0.449, and 0.1998 mmol/kg dw. The XO inhibitory activities of four mushrooms were assayed, with the IC_50_ values of 39.97, 20.71, 11.71, and 23.85 μg/mL, respectively. *L. nuda* and *T. terreum* may be effective for hyperuricemia and gout, which is associated with the results of phenolic compounds, some vitamins, trace elements, and linoleic acid contents on the inhibitory activities against xanthine oxidase. The results of medicinal mushrooms have shown that they could potentially be useful as inhibitors for the prevention of XO‐related diseases induced by ROS.

## Graphical abstract

In the current study, extracts from the fruiting bodies of four edible mushrooms were evaluated for chemical composition, antioxidant, and antiradical properties in various assay systems, specifically for total antioxidant activity. Total phenolic assay and total flavonoids, anti‐hemolytic activity, superoxide, ABTS, DPPH radical scavenging activity, inhibitory effects of the extracts on the xanthine oxidase enzyme, in addition to their quantification of phenolic compounds, identification of fatty acids, trace elements, mineral, and vitamin contents. *Lepista nuda* and *Tricholoma terreum* may be effective for hyperuricemia and gout, which is associated with the results of total phenolic compounds, vitamins, and linoleic acid contents on the inhibitory activities against xanthine oxidase. The results of medicinal mushrooms have shown that they could potentially be useful as inhibitors for the prevention of XO‐related diseases induced by ROS.

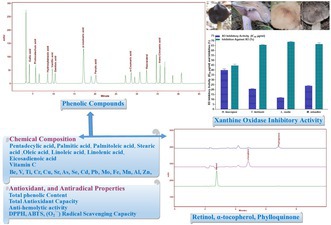

## 1 Introduction

Natural compounds present in food and medicine have attracted increasing attention due to their multiple health benefits and minimal side effects, which has led to their growing acceptance as dietary ingredients (Wang et al. 2024). Edible mushrooms have a high nutritional value and contain high‐quality proteins, dietary fiber, multivitamins, antioxidant phenolic compounds, and minerals. They also have low levels of unsaturated fatty acids, which makes them a nutritious choice (Zheng et al. 2025). The present study focuses on mushrooms, which are an important source of antioxidant compounds such as trace elements (Zn and Se), vitamins (retinol, and α‐tocopherol), and phenolic compounds. The delicate taste and texture of the four edible mushrooms: *Helvella leucopus* Pers., *Tricholoma terreum* (Schaeff.) P. Kumm, *Marasmius oreades* (Bolton) Fr, and *Lepista nuda* (Bull.) Cooke are consumed by many people and, at the same time, used in traditional medicine.

Reactive oxygen species (ROS) can trigger various harmful oxidative processes. The relationship between inflammation and cancer involves ROS that are secreted by phagocytic cells. Excessive and sustained production of ROS by inflammatory cells is considered a major factor in their genotoxic effects. The formation of ROS in the cells is associated with several physiological functions, such as the activation of NAD(P)H oxidase, xanthine oxidase (XO) and the respiratory chain in the cellular mitochondria (Lin et al. 2008). The complex molybdoflavoprotein known as XO (EC1.2.3.2) occurs primarily in the dehydrogenase form (XD); just the oxidase type of the enzyme is linked to the production of significant quantities of hydrogen peroxide and superoxide. XO increases the level of oxidative stress in an organism by catalyzing the oxidation of hypoxanthine and xanthine to uric acid, which produces superoxide radicals. Gout is due to high levels of uric acid in the blood or hyperuricemia (Ozyurek et al. 2009). Allopurinol is an XO inhibitor used clinically to treat gout, but it has numerous side effects, including nephropathy, allergic reactions, and hepatitis. It is therefore preferable to develop new alternatives with a greater therapeutic effect and fewer adverse effects (Nguyen et al. 2004).

This study aimed to evaluate the antioxidant properties and chemical composition of methanol extracts of four edible mushrooms: *H. leucopus* Pers., *T. terreum* (Schaeff.) P. Kumm, *Marasmius oreades* (Bolton) Fr., and *L. nuda* (Bull.) Cooke. Elemental analyses were conducted using ICP‐OES. Anti‐hemolytic activity, superoxide, ABTS, and DPPH radical scavenging activity were measured using a UV–visible spectrophotometer. Quantification of phenolic compounds and vitamins (α‐tocopherol, all‐trans‐retinol, and phylloquinone) was performed by high‐performance liquid chromatography (HPLC), while detection of fatty acids was determined by gas chromatography–mass spectrometry (GC–MS). In addition, four mushroom extracts were evaluated to determine the inhibitory activities against XO.

## 2 Materials and Methods

### 2.1 Mushroom Material

Fruiting bodies of four edible mushrooms *H. leucopus* Pers. (under *Pinus sylvestris* L. trees, 19.05.2021, 40°23.725′ N, 42°38.447′ E, 2010 m.), *T. terreum* (Schaeff.) P. Kumm. (under *P. sylvestris* L. trees, 29.05.2021, 40°21.996′ N, 42°31.772′ E, 2265 m.), *L. nuda* (Bull.) Cooke (under *P. sylvestris* L. trees, 11.06.2022, 40°19.156′ N, 42°32.520′ E, 2155 m.) and *M. oreades* (Bolton) Fr. (under *P. sylvestris* L. trees, 12.06.2022, 40°19.875′ N, 42°32.562′ E, 2157 m.) used in the study were collected at Kars, Sarıkamış district, Türkiye, during May–June of 2021–2022. Four species were identified and classified by a mycologist Dr. Emre AKCAY, University of Van Yüzüncü Yil, Science Faculty, Biology Department. Witness mushroom specimens, deposited with the code (Fungarium codes MEA. 98, MEA. 615, MEA. 644 and MEA. 743, respectively) in Van Yüzüncü Yil University, Science Faculty, at Fungarium.

### 2.2 Determination of α‐Tocopherol, Retinol, and Phylloquinone

#### 2.2.1 Standard Solutions and Calibration

**FIGURE 1:**
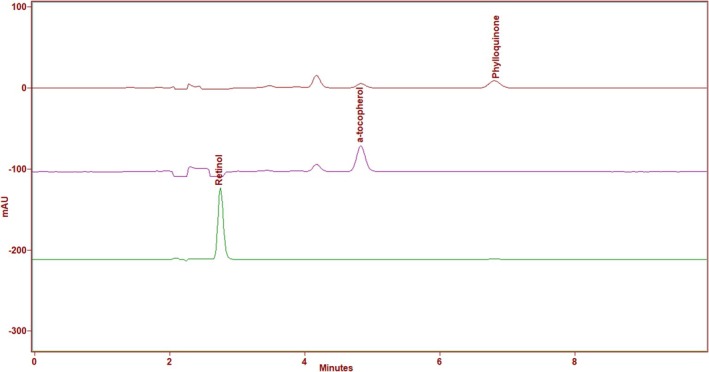
HPLC chromatogram of retinol, α‐tocopherol, and phylloquinone at 325, 290, 248 nm.

Stock solutions of phylloquinone, all‐trans‐retinol, and α‐tocopherol were established at 500 μg/mL in methanol. To prepare the standards, stock solutions were appropriately diluted with the mobile phase according to the appropriate ratio. The ranges of the standards for the vitamins (0.2–2 μg/mL). A linear regression analysis was conducted to compare the concentrations of the standard solutions with the peak area to determine the calibration (Figure 1).

#### 2.2.2 Extraction Procedure

We modified the method by Sahin et al. (2005) and Al‐Saleh et al. (2006) to assess the quantities of all‐trans‐retinol, α‐tocopherol, and phylloquinone in *H. leucopus, T. terreum, L. nuda*, and *M. oreades* . Five grams were weighed and extracted using *n*‐hexane and ethanol from the dried and ground‐in‐the‐shade mushroom samples. They were mixed with 0.01% BHT, vortexed, and left in the dark for a whole day. Then, they were centrifuged for 10 min. at 4000 rpm and + 4°C. After filtering the supernatant using Whatman filter paper, 0.5 mL of n‐hexane was then added, and the mixture was dried at 37°C using nitrogen gas. Following drying, the precipitate was prepared for analysis by dissolving it in a 200 μL methanol + tetrahydrofuran.

#### 2.2.3 Chromatographic Conditions

Gl Science C_18_ reversed‐phase HPLC column (250 × 4.6 mm ID) with a mobile phase of methanol and tetrahydrofuran (80:20) was used at a temperature of 25°C and a flow rate of 1.5 mL/min. The use of a PDA array detector, applications in a 100‐μL volume in dark vials in a tray autosampler (−8°C) were at 325, 290, and 248 nm (α‐tocopherol, all‐trans‐retinol, and phylloquinone) in Thermo Scientific Finnigan Surveyor HPLC. Isocratic elution was used in the chromatographic determination, which was performed at 40°C.

### 2.3 Vitamin C Measurement

A stock solution of vitamin C containing 4000 mg/mL was established in metaphosphoric acid. The standard solution was prepared by diluting the stock solutions appropriately with double‐distilled water. The calibration was determined by linear regression analysis comparing the absorbance with the concentration of the standard solution. The 2,4‐dinitrophenylhydrazine technique was used to determine the vitamin C content of *H. leucopus, T. terreum, L. nuda*, and *M. oreades*.

### 2.4 Element Determination

The dry ashing method was used to determine the mineral content of *H. leucopus, T. terreum, L. nuda*, and *M. oreades* . One gram of the sample was weighed, put into the crucible, and oven to 105° for 4–5 h. The dried samples were then crushed in carefully cleaned porcelain mortars. For each sample, 2 mL of a 95:5 ethyl alcohol‐sulfuric acid mixture was transferred. The samples were then placed in a 250°C ash furnace. The temperature of the furnace was increased by 100°C per hour until it reached 550°C. The samples collected from the ash furnace were mixed with 5 mL of prepared 3N hydrochloric acid solution and then mixed with 50 mL of distilled water. The metal analysis was performed using multi‐element reference materials (NIST SRM) (Inorganic Ventures IV‐Stock‐1643). The elemental analysis of Be, V, Ti, Cr, Cu, Sr, As, Se, Cd, Pb, Mo, Fe, Mn, Al, Zn, and K was performed using ICP‐OES. The analyses were carried out using ICP‐OES (Thermo ICP‐OES iCAP 6300 Duo, England) and an ASX‐520 autosampler.

### 2.5 Antioxidant Screening

#### 2.5.1 Extraction Procedure

The fruiting bodies of the mushroom samples were separated and carefully removed from dust and impurities. The mushroom samples of the fruiting bodies were air‐dried in the shade without exposure to sunlight at room temperature using blotter paper and then stored for analysis. After the mushroom was dried in the shade and ground into powder, 20 g of the powdered mushroom samples were weighed and then transferred to a colored bottle to which 0.4 L of 75% methanol was added. The sample preserved in the colored bottle was extracted for 48 h at room temperature with stirring. The solution was then separated from the residue by filtration. The methanol in the filtrate was eliminated with a rotary evaporator, and the filtrate was prepared by freeze‐drying (−86°C).

#### 2.5.2 Analysis of the Total Phenol Content

The reagent (Folin–Ciocalteu) was prepared to analyze the total phenolic content of *H. leucopus, T. terreum, L. nuda*, and *M. oreades* (Yi et al. 1997; Gamez‐Meza et al. 1999). After the mushroom samples were diluted with methanol, 0.3 mL of 20% Na_2_CO_3_ was added. Next, 100 μL of water‐diluted Folin reagent (1:1) was added to the mixture, and the samples were incubated for 2 h at room temperature. Sample absorbance was measured at a wavelength of 765 nm in comparison to a control sample. Gallic acid equivalents (mg GAE/g) is a unit of measurement used to express phenolic compound concentration in an extract.

#### 2.5.3 Analysis of Total Antioxidant Capacity

The total antioxidant capacity of *H. leucopus, T. terreum, L. nuda*, and *M. oreades* was determined in this work using the methodology developed by Prieto et al. (1999). The assay is based on the formation of the green phosphate/Mo(V) complex, which results from the reduction of acidic Mo(VI) to (V) at acidic pH. 0.2 mL of reagents (0.6 M, 28 mM, and 4 mM sodium phosphate, ammonium molybdate, and sulfuric acid) was added to 0.2 mL samples of various amounts of methanol‐diluted mushroom extract, and the samples were then stored at 95°C for 90 min. The samples were cooled to room temperature before the absorbance of the samples was measured at a wavelength of 695 nm in comparison with the control sample. The antioxidant capacity was expressed as mM ascorbic acid/g.

#### 2.5.4 DPPH Radical Scavenging Capacity

The DPPH radical scavenging assay is based on the spectrophotometric measurement of the characteristic purple lightening caused by these chemicals scavenging the stable DPPH free radical in the presence of antioxidant chemicals that donate hydrogen atoms or electrons (Chen et al. 2009; Cuendet et al. 1997). The methanol‐diluted different concentrations of the mushrooms (*H. leucopus, T. terreum, L. nuda*, and *M. oreades* ) extract were subjected to an incubation period of 30 min, after which the absorbance at 517 nm was determined against a blank. The graph showing the percentage inhibition versus extract concentration was used to calculate the IC_50_ values or μg/mL required to inhibit the generation of DPPH radicals by 50%, which were used to express the antioxidant activity of the samples. The positive control was BHT. The % inhibition of DPPH free radical was determined using the following formula.

$$\text{Inhibition}\;\left(\%\right)=\left\{\frac{A_{\text{Blank}}-A_{\text{Sample}}}{A_{\text{Blank}}}\right\}\times 100$$

#### 2.5.5 ABTS Radical Scavenging Capacity

*Helvella leucopus, T. terreum, L. nuda*, and *M. oreades* were used in this study to evaluate the radical scavenging activity of ABTS. The 0.1 M phosphate buffer with pH 7.4 was used, and 2 mM ABTS solution and 2.45 mM potassium persulfate solution were incubated in the dark for 16 h at room temperature. The absorbance of the resulting solution was recorded at 734 nm. Trolox, a synthetic antioxidant, was used to test the ability of molecules to scavenge stable free radicals (Arnao et al. 2001).

$$\text{Inhibition}\;\left(\%\right)=\left\{\frac{A_{\text{Blank}}-A_{\text{Sample}}}{A_{\text{Blank}}}\right\}\times 100$$

#### 2.5.6 Phenylhydrazine Method

In this procedure, 1.85 mL of buffer, 0.1 mL of 20% PCV, and 1 mL of phenylhydrazine were mixed with samples prepared with various concentrations of the methanol extract of the four species of mushrooms. They were then incubated at 37°C for 1 h before being centrifuged at 4000 rpm for 10 min. The supernatant fraction was then collected into separate tubes, and the absorbance at 540 nm was compared with the sample used as a control (Valenzuela et al. 1985).

#### 2.5.7 Superoxide Anion Radical Scavenging Activity

The superoxide radical scavenging activity of the four mushroom extracts was evaluated using a modified version of the technique of Robak and Gryglewski (1988) and Lee et al. (2002). Briefly, 0.1 M potassium phosphate buffer (pH 7.4) was used to dissolve xanthine (2 nM) and NBT (12 nM) to prepare the solution. The mixture was then incubated at 37°C for 10 min. A spectrophotometer was used to measure the blue color developed at 560 nm. The superoxide anion radical scavenging activity was determined using the following formula.

$$\begin{array}{c} \text{Superoxide anion radical scavenging activity}\;\left(\%\right)\\ =\left\{\frac{A_{\text{Blank}}-A_{\text{Sample}}}{A_{\text{Blank}}}\right\}\times 100 \end{array}$$

The superoxide radical scavenging activity of the sample was reported as the inhibition plot calculated IC_50_ values (μg/mL) required to inhibit superoxide radical production by 50%.

### 2.6 Mushroom Samples and Extraction for Phenolics

The mushroom fruiting bodies were cut into small pieces and air‐dried in an oven at 40°C. After fine drying (20 mesh) of dried mushroom samples weighing 10 g, 100 mL of methanol was added and stirred for 24 h at 30°C and 120 rpm. The resulting filtrate was then filtered through Whatman No. 1 filter paper and collected. The filtration residue was removed. The mixed methanol extracts were obtained with an evaporator (IKA RV 10) at 37°C. The dry extract was then lyophilized using a freeze dryer at −86°C.

#### 2.6.1 Quantification of Phenolics Using HPLC

**FIGURE 2:**
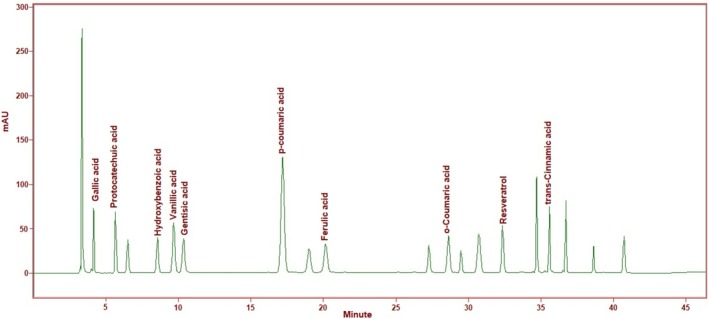
The gallic acid, protocatechuic acid, hydroxybenzoic acid, vanillic acid, gentisic acid, *p*‐coumaric acid, ferulic acid, o‐coumaric acid, resveratrol, trans‐cinnamic acid chromatograms.

HPLC was applied to study the phenolic extract. For each sample corresponding to the treatments, the following phenolic compounds were measured: Gallic acid, protocatechuic acid, vanillic acid, gentisic acid, hidroksibenzoic acid, *p*‐coumaric acid, ferulic acid, *o*‐coumaric acid, resveratrol, and trans‐cinnamic acid. Using the Agilent 1260 Infinity HPLC‐DAD and an integrated system, the composition of phenolic compounds in different species of mushrooms was analyzed using HPLC equipment with an injection volume adjustment of 10 μL. A column (4.6 × 250 mm, 5 μm; ACE Generix C18 [GEN‐7444], Scotland) thermostated at 30°C was used for chromatographic separation. A flow rate of 0.8 mL per minute was used. The mobile phases were (A) water containing 0.1% phosphoric acid and (B) 100% HPLC‐grade acetonitrile. By comparing the peaks observed at 300 and 200 nm with the standard curves of each phenolic based on a calibration curve, the quantities of phenolic compounds were measured. By comparing their retention times with those of reference compounds, phenolics can be distinguished. The wavelength chosen for detection in a DAD was recorded at 300 nm, and the results were expressed as μg per gram of dry weight (dw) (Figure 2).

### 2.7 Identification of Fatty Acids

The identification of volatile compounds of *H. leucopus, T. terreum, L. nuda*, and *M. oreades* was performed by using GC–MS. A gas chromatograph instrument with a split/splitless injector and an FID and MS (GC–MS, Agilent 7890 GC/5970 MS Series‐Santa Clara, CA, USA). Fatty acids were measured after a methyl esterification with modifications procedure as previously reported by the authors (Folch et al. 1957; Hara and Radin 1978). The fatty acids were detected by comparing the relative retention times of the FAME peaks of the samples with the standards. The software with the NIST and WHILEY libraries registered in the system recorded the results. The results were presented as relative percentages of the different fatty acids.

### 2.8 Determination of Xanthine Oxidase Inhibitory Activity

The spectrophotometric evaluation of uric acid production from xanthine was used to determine, with some minor modifications, the XO inhibitory activity of the mushroom samples. The absorbance of the incubation solution was determined at 295 nm in comparison to a blank without enzyme. For each concentration, various sample concentrations were dissolved in a pH 7.4 potassium phosphate buffer solution. The effect on uric acid production allowed the calculation of IC_50_ values in regression analysis. A buffer was used as a blank, and a solution consisting of xanthine and XO was used as a control. Allopurinol was used as a positive control. The percentage inhibition of XO activity was determined using the following formula (Candan 2003; Ozyurek et al. 2009).

$$\text{Inhibition}\;\left(\%\right)=\left\{\frac{A_{\text{Control}}-A_{\text{Sample}}}{A_{\text{Control}}}\right\}\times 100$$

### 2.9 Statistical Method and Analysis

The results are reported as mean values and standard errors of mean ($\bar{X}$ ± SEM). To assess the correlations among variables, Pearson correlation analysis was used to examine for significant relationships between XO inhibitory activity (XO‐IC_50_) and parameters of four mushroom species (*T. terreum, H. leucopus, L. nuda*, and *M. oreades* ). The statistical analysis was performed with descriptive analysis and correlation by using a statistical program (SPSS 22). A nonlinear regression analysis was carried out to calculate the IC_50_ values.

## 3 Results

The presence of gallic acid, protocatechuic acid, hydroxybenzoic acid, vanillic acid, ferulic acid, *o*‐coumaric acid, resveratrol, and trans‐cinnamic acid was investigated in the four edible mushrooms fruiting bodies of extract by using the HPLC procedure. The gentisic acid and *p*‐coumaric acid have not been identified in the four edible mushrooms. The quantification of phenolic compounds levels is given in Table 1.

**TABLE 1:** Phenolic compounds of *Helvella leucopus, Tricholoma terreum, Lepista* *nuda*, and *Marasmius oreades* mushroom species.

μg/g dw	*Helvella leucopus* ($\bar{X}$ ± SEM)	*Tricholoma terreum* ($\bar{X}$ ± SEM)	*Lepista nuda* ($\bar{X}$ ± SEM)	*Marasmius oreades* ($\bar{X}$ ± SEM)
Gallic acid: *y* = 16.5429 + 15.10588*x*; *r* ^2^ = 0.97624	133.04 ± 25.012	246.49 ± 7.236	408.64 ± 15.782	129.302 ± 49.18
Protocatechuic acid: *y* = 3.94086 + 27.67447*x*; *r* ^2^ = 0.99976	1.569 ± 0.414	ND	ND	ND
Hydroxybenzoic acid: *y* = 2.91111 + 23.70587*x*; *r* ^2^ = 0.99982	1.0065 ± 0.259	0.3205 ± 0.015	2.244 ± 0.417	ND
Vanillic acid: *y* = 4.69076 + 39.2259*x*; *r* ^2^ = 0.99979	0.274 ± 0.0739	0.0890 ± 0.0685	0.0892 ± 0.0259	ND
Gentisic acid: *y* = 3.5651 + 28.24135*x*; *r* ^2^ = 0.99958	ND	ND	ND	ND
*p*‐coumaric acid: *y* = 14.09979 + 127.29898*x*; *r* ^2^ = 0.9999	ND	ND	ND	ND
Ferulic acid: *y* = 4.61719 + 31.9296*x*; *r* ^2^ = 0.9996	ND	ND	0.622 ± 0.0428	ND
*o*‐coumaric acid: *y* = 3.75034 + 31.31551*x*; *r* ^2^ = 0.99965	9.442 ± 1.293	2.766 ± 0.152	3.216 ± 0.398	1.310 ± 0.469
Resveratrol: *y* = 2.42681 + 31.75884*x*; *r* ^2^ = 0.99988	0.169 ± 0.0727	0.155 ± 0.009	ND	ND
Trans‐cinnamic acid: *y* = 2.60643 + 24.9521*x*; *r* ^2^ = 0.99972	0.2061 ± 0.1399	ND	3.819 ± 0.799	2.872 ± 0.395
Total ($\bar{X}$ ± SEM)	20.82 ± 3.89	49.97 ± 1.49	69.77 ± 2.91	44.49 ± 16.68
Total	145.71 ± 27.26	249.82 ± 7.48	418.63 ± 17.47	133.48 ± 50.046

Abbreviation: ND, not detected.

Trace elements (Be, V, Ti, Cr, Cu, Sr, As, Se, Cd, Pb, Mo, Fe, Mn, Al, Zn) and mineral (K) concentrations were determined by ICP‐OES in parts of the fruiting body of four edible mushrooms, *H. leucopus, T. terreum, L. nuda*, and *M. oreades* . The finding of major and trace mineral, total antioxidant capacity, total phenolic, retinol, α‐tocopherol, phylloquinone, and ascorbic acid contents of *H. leucopus, T. terreum, L. nuda*, and *M. oreades* are shown in Table 2.

**TABLE 2:** α‐tocopherol, retinol, phylloquinone, ascorbic acid, total antioxidant capacity, total phenolic, element (Be, V, Ti, Cr, Cu, Sr, As, Se, Cd, Pb, Mo, Fe, Mn, Al, Zn, and K) content in *leucopus, Tricholoma terreum, Lepista nuda*, and *Marasmius oreades* mushroom species.

	*Helvella leucopus* ($\bar{X}$ ± SEM)	*Tricholoma terreum* ($\bar{X}$ ± SEM)	*Lepista nuda* ($\bar{X}$ ± SEM)	*Marasmius oreades* ($\bar{X}$ ± SEM)
α‐tocopherol (μmol/kg)	0.6009 ± 0.139	0.79 ± 0.0064	0.3581 ± 0.052	ND
Retinol (μmol/kg)	0.058 ± 0.003	0.0473 ± 0.001	0.048 ± 0.0007	ND
Phylloquinone (μmol/kg)	ND	0.1162 ± 0.025	0.208 ± 0.0121	0.166 ± 0.0058
Ascorbic A. (mg/100 g)	31.886 ± 3.157	59.511 ± 7.103	296.290 ± 48.145	7.419 ± 0.789
T. phenolic c (mg GA/g)	2.59 ± 0.079	3.18 ± 0.451	2.65 ± 0.027	2.76 ± 0.027
Total antioxidant capacity (mM A.A/g)	22.150 ± 0.569	12.755 ± 0.854	24.712 ± 1.424	10.477 ± 1.993
Be (μmol/kg)	1.118 ± 0.0431	1.0243 ± 0.073	0.931 ± 0.0521	0.817 ± 0.0742
V (μmol/kg)	3.395 ± 0.687	11.805 ± 0.195	15.0915 ± 1.590	7.023 ± 0.569
Ti (μmol/kg)	11.153 ± 1.578	7.7684 ± 1.893	40.587 ± 3.480	17.519 ± 7.789
Cr (μmol/kg)	4.019 ± 0.116	1.419 ± 0.0515	3.781 ± 0.301	1.564 ± 0.909
Cu (μmol/kg)	71.79 ± 19.804	27.931 ± 0.253	78.167 ± 1.3802	53.348 ± 6.143
Sr (μmol/kg)	9.474 ± 0.386	2.703 ± 0.0254	3.288 ± 0.552	3.590 ± 0.171
As (μmol/kg)	2.363 ± 0.001	0.449 ± 0.053	1.213 ± 0.189	0.409 ± 0.0483
Se (μmol/kg)	0.623 ± 0.091	0.328 ± 0.109	0.449 ± 0.189	0.295 ± 0.0127
Cd (μmol/kg)	1.575 ± 0.055	5.533 ± 0.089	0.684 ± 0.0151	0.0532 ± 0.006
Pb (μmol/kg)	0.097 ± 0.042	0.1402 ± 0.018	0.240 ± 0.0312	0.101 ± 0.0429
Mo (μmol/kg)	0.0679 ± 0.024	0.0147 ± 0.002	0.0717 ± 0.0077	0.0214 ± 0.0118
Fe (mmol/kg)	0.248 ± 0.021	0.237 ± 0.0204	0.449 ± 0.0279	0.1998 ± 0.0469
Mn (μmol/kg)	11.318 ± 0.332	26.034 ± 2.482	48.362 ± 1.359	45.248 ± 0.494
Al (μmol/kg)	3.928 ± 0.363	7.298 ± 0.104	9.085 ± 0.599	4.712 ± 0.379
Zn (μmol/kg)	309.064 ± 6.55	108.825 ± 0.21	118.715 ± 0.108	93.768 ± 2.542
K (mmol/kg)	1.634 ± 0.002	1.883 ± 0.0389	1.719 ± 0.035	1.621 ± 0.0105

*Note:* Values are expressed as mean ± SE of the mean ($\bar{X}$ ± SEM). Samples were carried out in triplicate.

The results of the pentadecylic acid, palmitic acid, stearic acid, palmitoleic acid, oleic acid, linolenic acid, linoleic acid, eicosadienoic acid, as also their monounsaturated fatty acids (MUFA), polyunsaturated fatty acids (PUFA), and saturated fatty acids (SFA) percentages are also presented in Table 3.

**TABLE 3:** Fatty acid composition (%) of the *leucopus, Tricholoma terreum, Lepista nuda,* and *Marasmius oreades* mushroom species.

	Carbon number	*Helvella leucopus* (%)	*Tricholoma terreum* (%)	*Lepista nuda* (%)	*Marasmius oreades* (%)
Pentadecylic acid	C15:0	ND	5.57	6.80	ND
Palmitic acid	C16:0	18.05	18.78	22.28	30.30
Palmitoleic acid	C16:1	1.83	4.19	ND	6.31
Stearic acid	C18:0	11.33	13.51	17.13	22.92
Oleic acid	C18:1	35.37	36.31	23.41	19.61
Linoleic acid	C18:2	30.82	19.92	30.38	20.86
Linolenic acid	C18:3	ND	1.72	ND	ND
Eicosadienoic acid	C20:2	2.60	ND	ND	ND
ΣSFA		29.38	37.86	46.21	53.22
ΣMUFA		37.19	40.50	23.41	25.92
ΣPUFA		33.42	21.64	30.38	20.86
ΣUFA		70.62	62.14	53.79	46.78

Abbreviations: MUFA, monounsaturated fatty acids; ND, not detected; PUFA, polyunsaturated fatty acids; SFA, saturated fatty acids; UFA, unsaturated fatty acid.

Anti‐hemolytic activity, superoxide, DPPH, ABTS, radical scavenging activities of *H. leucopus, T. terreum, L. nuda* and *M. oreades* methanol extracts along with the standard reference BHT, and trolox are shown in Table 4.

**TABLE 4:** Values of % Inhibition and IC_50_ (μg/mL) in methanol extracts of *leucopus, Tricholoma terreum*, *Lepista nuda*, and *Marasmius oreades* mushroom species, compared with a positive controls.

	% Inhibition $\bar{X}$ ± SEM	IC_50_ (μg/mL) $\bar{X}$ ± SEM
*Helvella leucopus*		
DPPH^•^	57.531 ± 0.247	45.562 ± 1.126
ABTS	73.397 ± 0.801	30.011 ± 2.548
PhNHNH_2_	57.18 ± 0.15	33.68 ± 1.43
Superoxide anion	65	124.977
*Tricholoma terreum*		
DPPH^•^	53.981 ± 0.401	44.619 ± 2.315
ABTS	66.031 ± 1.336	51.622 ± 0.745
PhNHNH_2_	57.36 ± 0.25	35.34 ± 1.75
Superoxide anion	73	12.89234
*Lepista nuda*		
DPPH^•^	62.037 ± 0.370	50.535 ± 1.029
ABTS	65.935 ± 0.668	44.892 ± 1.029
PhNHNH_2_	58.36 ± 2.23	37.09 ± 1.54
Superoxide anion	72	32.70621
*Marasmius oreades*		
DPPH^•^	53.055 ± 0.586	89.321 ± 2.37
ABTS	58.333 ± 0.801	65.062 ± 0.365
PhNHNH_2_	62.08 ± 2.28	51.88 ± 2.53
Superoxide anion	58	77.04739
Control		
DPPH/BHT	71.11 ± 0.694	17.064 ± 1.517
ABTS/Trolox	83.696 ± 0.544	27.464 ± 0.212
PhNHNH_2_/Trolox	51.14 ± 0.10	17.24 ± 0.54
Superoxide anion/BHT	NT	NT

Abbreviation: NT, not tested.

## 4 Discussion

In the current study, extracts from the fruiting bodies of four edible mushrooms were evaluated for chemical composition, antioxidant, and antiradical properties in various assay systems, specifically for total antioxidant activity, total phenolic assay, anti‐hemolytic activity, superoxide, ABTS, DPPH radical scavenging activity, and inhibitory effects of the extracts on the XO enzyme. In addition to their quantification of phenolic compounds, identification of fatty acids, trace elements, mineral, and vitamin contents was conducted.

Mushrooms are often used in medicine, pharmacy, cosmetics, and commerce due to their beneficial metabolic content. Known as a great source of nutrition, macro mushrooms are rich in protein, fiber, minerals, vitamins, and many different nutrients, while being low in carbohydrates and fat (Selem et al. 2021). Mushrooms generate different varieties of secondary metabolites with potent biological roles and possess the potential to be used as antitumor, antioxidant, immunomodulatory, cardiovascular, antihyperlipidemic, antibacterial, antihypercholesterolemic, antiviral, antifungal, antiparasitic, antidiabetic, and hepatoprotective drugs (Im et al. 2016).

Phenolic compounds with one or more hydroxyl groups are able to exhibit antioxidant properties against free radicals or transition metals in oxidation reactions (Ceylan et al. 2021). Phenolics and flavonoids can also act as effective inhibitors against metabolic enzymes that include XO, cyclooxygenase, and lipoxygenase. Therefore, phenols and flavonoids may have a major role in the inhibition of XO (Nile et al. 2018). XO is a flavoprotein that catalyzes the oxidation of hypoxanthine to xanthine and produces superoxide and uric acid. XO inhibitors have been demonstrated to be effective in treating liver disease and gout induced by the generation of uric acid and superoxide anion radicals (Sahgal et al. 2009). Uric acid is not subject to any further metabolism in humans and is excreted via the kidneys and intestines. Accordingly, higher uric acid concentrations may be a reaction to increased XO activity and oxidative stress, which are characteristic of many vascular diseases (Kostić et al. 2015). Effective dietary XO inhibitors may reduce XO activity in vivo, potentially lowering hyperuricemia and oxidative stress (Dew et al. 2005).

**FIGURE 3:**
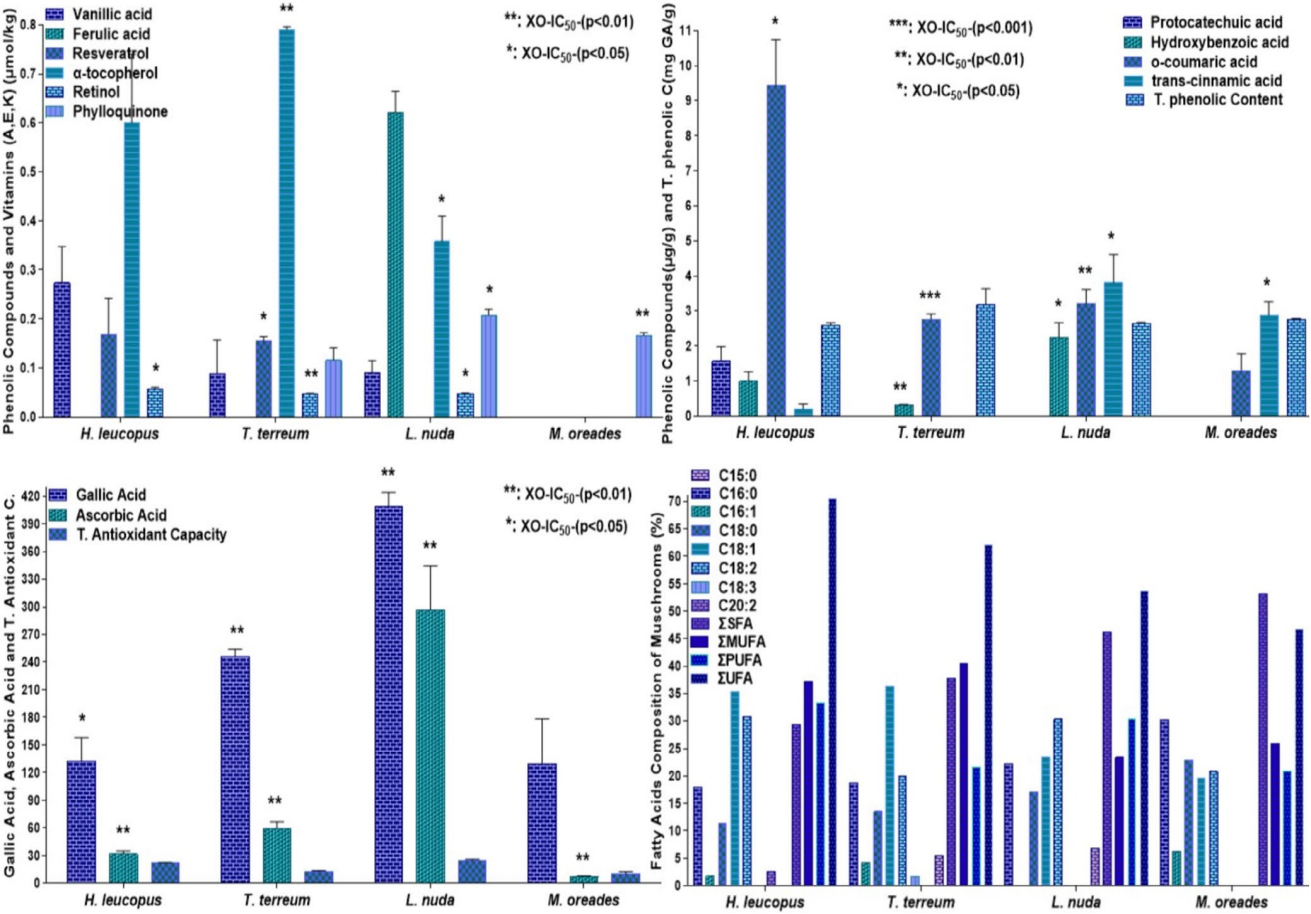
The gallic acid, protocatechuic acid, hydroxybenzoic acid, vanillic acid, ferulic acid, o‐coumaric acid, resveratrol, trans‐cinnamic acid, retinol, α‐tocopherol, phylloquinone, ascorbic acid, total antioxidant capacity, and total phenolic levels and the pentadecylic acid, palmitic acid, stearic acid, palmitoleic acid, oleic acid, linolenic acid, linoleic acid, eicosadienoic acid, MUFA, PUFA and SFA percentages. The values are given as mean ± standard error of the mean ($\bar{X}$ ± SEM). Significant association between XO inhibitory activity (XO‐IC_50_) and parameters (*p < 0.05, **p < 0.01, ***p ≤ 0.001).

Gallic acid, hydroxybenzoic acid, protocatechuic acid, vanillic acid, *o*‐coumaric acid, resveratrol, and trans‐cinnamic acid contents in *H. leucopus* extract were found to be 133.044 ± 25.012, 1.569 ± 0.414, 1.0065 ± 0.259, 0.274 ± 0.0739, 9.442 ± 1.293, 0.169 ± 0.0727, and 0.2061 ± 0.1399 μg/g, respectively. Gallic acid, hydroxybenzoic acid, vanillic acid, *o*‐coumaric acid, and resveratrol levels in *T. terreum* extract were determined to be 246.493 ± 7.236, 0.3205 ± 0.015, 0.0890 ± 0.0685, 2.766 ± 0.152, and 0.155 ± 0.009 μg/g, respectively. Gallic acid, hydroxybenzoic acid, vanillic acid, ferulic acid, *o*‐coumaric acid, and trans‐cinnamic acid values in *L. nuda* extract were observed to be 408.638 ± 15.782, 2.244 ± 0.417, 0.0892 ± 0.0259, 0.622 ± 0.0428, 3.216 ± 0.398, and 3.819 ± 0.799 μg/g, respectively. Gallic acid, *o*‐coumaric acid, and trans‐cinnamic acid concentrations in *M. oreades* extract were detected to be 129.302 ± 49.182, 1.310 ± 0.469, and 2.872 ± 0.395 μg/g, respectively, on a dry weight basis (Table 1, Figure 3).

When the results were evaluated, it was detected that four edible mushrooms fruiting bodies of values of gallic acid, *o*‐coumaric acid, and trans‐cinnamic acid had a significantly high level. Only a small quantity of vanillic acid was determined. In this study, it was found that the phytochemical constituents of methanol extracts in the four mushrooms have found the presence of gallic acid, *o*‐coumaric acid, and trans‐cinnamic acid linked to their antioxidant capacity and antitumor activities. *L. nuda* contains the analyzed total phenolic compounds at the highest level (418.63 ± 17.47 μg/g) and this is followed by *T. terreum* (249.82 ± 7.48 μg/g), *H. leucopus* (145.71 ± 27.26 μg/g) and *M. oreades* (133.48 ± 50.046 μg/g).

In an HPLC study conducted by Dundar et al., the protocatechuic acid, vanillic acid, *p*‐coumaric acid, and *o*‐coumaric acid levels of the *H. leucopus mushroom* are found to be 4.36, 25.51, 0.31, and 297.65 mg/kg, respectively. It was determined that the phenolic compound levels were lower than those of the *H. leucopus* mushroom reported by (Dundar et al. 2015). In a different study, Erbiai et al. revealed that the gallic acid content was lower in the *L. nuda* samples measured by HPLC–MS, finding 131.7 ± 1.11 μg/g dw compared to this study's values. Furthermore, it is reported that the values of hydroxybenzoic acid, vanillic acid, and ferulic acid levels are 587.9 ± 4.89, 23.53 ± 1.10, and 27.3 ± 0.53 μg/g dw in the *L. nuda* , which are higher compared to this study's findings (Erbiai et al. 2023).

Certain polyphenolic compounds with chemical structures that are effective in scavenging free radicals have a higher antioxidant activity as compared to common antioxidants such as vitamins E and A (Wu et al. 2010). Gallic acid has previously been studied for its antioxidant, antifungal, anti‐inflammatory, antidiabetic, antibacterial, anticancer, and antiviral activities. *p*‐coumaric acid and its different conjugates have been investigated for their different biological activities, for example, antioxidant, antimicrobial, anticancer, anti‐inflammatory, antiviral, anti‐arthritis, anti‐hyperlipidemia, and antidiabetic activities (Altay et al. 2022). Vanillic acid, a derivative of benzoic acid, is used as a flavoring agent in the food, cosmetics, and pharmaceutical industries. It also has a number of different pharmacological activities, including antioxidant, anti‐apoptotic, anti‐inflammatory, hepatoprotective, cardioprotective, neuroprotective, and immunoprotective activities (Guven et al. 2023).

All metal levels were measured on a dry‐weight basis. Be, V, Ti, Cr, Cu, Sr, As, Se, Cd, Pb, Mo, Fe, Mn, Al, Zn, and K contents in *H. leucopus* were determined as 1.118 ± 0.0431 μmol/kg, 3.395 ± 0.687 μmol/kg, 11.153 ± 1.578 μmol/kg, 4.019 ± 0.116 μmol/kg, 71.79 ± 19.804 μmol/kg, 9.474 ± 0.386 μmol/kg, 2.363 ± 0.001 μmol/kg, 0.623 ± 0.091 μmol/kg, 1.575 ± 0.055 μmol/kg, 0.097 ± 0.042 μmol/kg, 0.0679 ± 0.024 μmol/kg, 0.248 ± 0.021 mmol/kg, 11.318 ± 0.332 μmol/kg, 3.928 ± 0.363 μmol/kg, 309.064 ± 6.55 μmol/kg, and 1.634 ± 0.002 mmol/kg. For *T. terreum*, values were found as 1.0243 ± 0.073 μmol/kg, 11.805 ± 0.195 μmol/kg, 7.7684 ± 1.893 μmol/kg, 1.419 ± 0.0515 μmol/kg, 27.931 ± 0.253 μmol/kg, 2.703 ± 0.0254 μmol/kg, 0.449 ± 0.053 μmol/kg, 0.328 ± 0.109 μmol/kg, 5.533 ± 0.089 μmol/kg, 0.1402 ± 0.018 μmol/kg, 0.0147 ± 0.002 μmol/kg, 0.237 ± 0.0204 mmol/kg, 26.034 ± 2.482 μmol/kg, 7.298 ± 0.104 μmol/kg, 108.825 ± 0.21 μmol/kg, and 1.883 ± 0.0389 mmol/kg. To *L. nuda* , concentrations were observed as 0.931 ± 0.0521 μmol/kg, 15.0915 ± 1.590 μmol/kg, 40.587 ± 3.480 μmol/kg, 3.781 ± 0.301 μmol/kg, 78.167 ± 1.3802 μmol/kg, 3.288 ± 0.552 μmol/kg, 1.213 ± 0.189 μmol/kg, 0.449 ± 0.189 μmol/kg, 0.684 ± 0.0151 μmol/kg, 0.240 ± 0.0312 μmol/kg, 0.0717 ± 0.0077 μmol/kg, 0.449 ± 0.0279 mmol/kg, 48.362 ± 1.359 μmol/kg, 9.085 ± 0.599 μmol/kg, 118.715 ± 0.108 μmol/kg, and 1.719 ± 0.035 mmol/kg. For *M. oreades* , levels were determined as 0.817 ± 0.0742 μmol/kg, 7.023 ± 0.569 μmol/kg, 17.519 ± 7.789 μmol/kg, 1.564 ± 0.909 μmol/kg, 53.348 ± 6.143 μmol/kg, 3.590 ± 0.171 μmol/kg, 0.409 ± 0.0483 μmol/kg, 0.295 ± 0.0127 μmol/kg, 0.0532 ± 0.006 μmol/kg, 0.101 ± 0.0429 μmol/kg, 0.0214 ± 0.0118 μmol/kg, 0.1998 ± 0.0469 mmol/kg, 45.248 ± 0.494 μmol/kg, 4.712 ± 0.379 μmol/kg, 93.768 ± 2.542 μmol/kg, and 1.621 ± 0.0105 mmol/kg, respectively.

Within the analyzed elements, K has the highest level, followed by Fe, Zn, Cu, and Mn in the mushroom sample. However, Mo and Pb were found to have the lowest metal concentrations. The sequence of element accumulation varies within the fruiting body when comparing the element accumulations of *H. leucopus*: K > Fe > Zn > Cu > Mn > Ti > Sr> Al > V > As > Cd > Be > Se > Pb > Mo. Comparison of the element abundance of *T. terreum* shows K > Fe > Zn > Cu > Mn > V > Ti > Al > Cd > Sr > As > Se > Pb > Mo. To compare the element concentration of *L. nuda* , K > Fe > Zn > Cu > Mn > Ti > V > Al > Cr > Sr > As > Be > Cd > Se > Pb > Mo. A comparison of the element values of *M. oreades* shows K > Fe > Zn > Cu > Mn > Ti > V > Al > Sr > Be > As > Se > Pb > Cd > Mo. Mushrooms may reduce the negative effects of free radicals because of their antioxidant properties. In the four edible mushrooms, high contents of Cu, Mn, and Zn are the co‐factors of antioxidant enzymes and can thus lead to the antioxidant potential of mushrooms. The findings show that the four edible mushrooms are mineral nutrients of choice.

The deposition of heavy metals in macrofungi has been revealed to be influenced by environmental and fungal factors. It is known that environmental factors such as organic matter content, metal concentration, pH in soil or substrate, and fungal factors including the fungal species, morphology of the fruit body, age of mycelium, developmental stages, and biochemical composition influence metal accumulation in macrofungi (Gebrelibanos et al. 2016).

**FIGURE 4:**
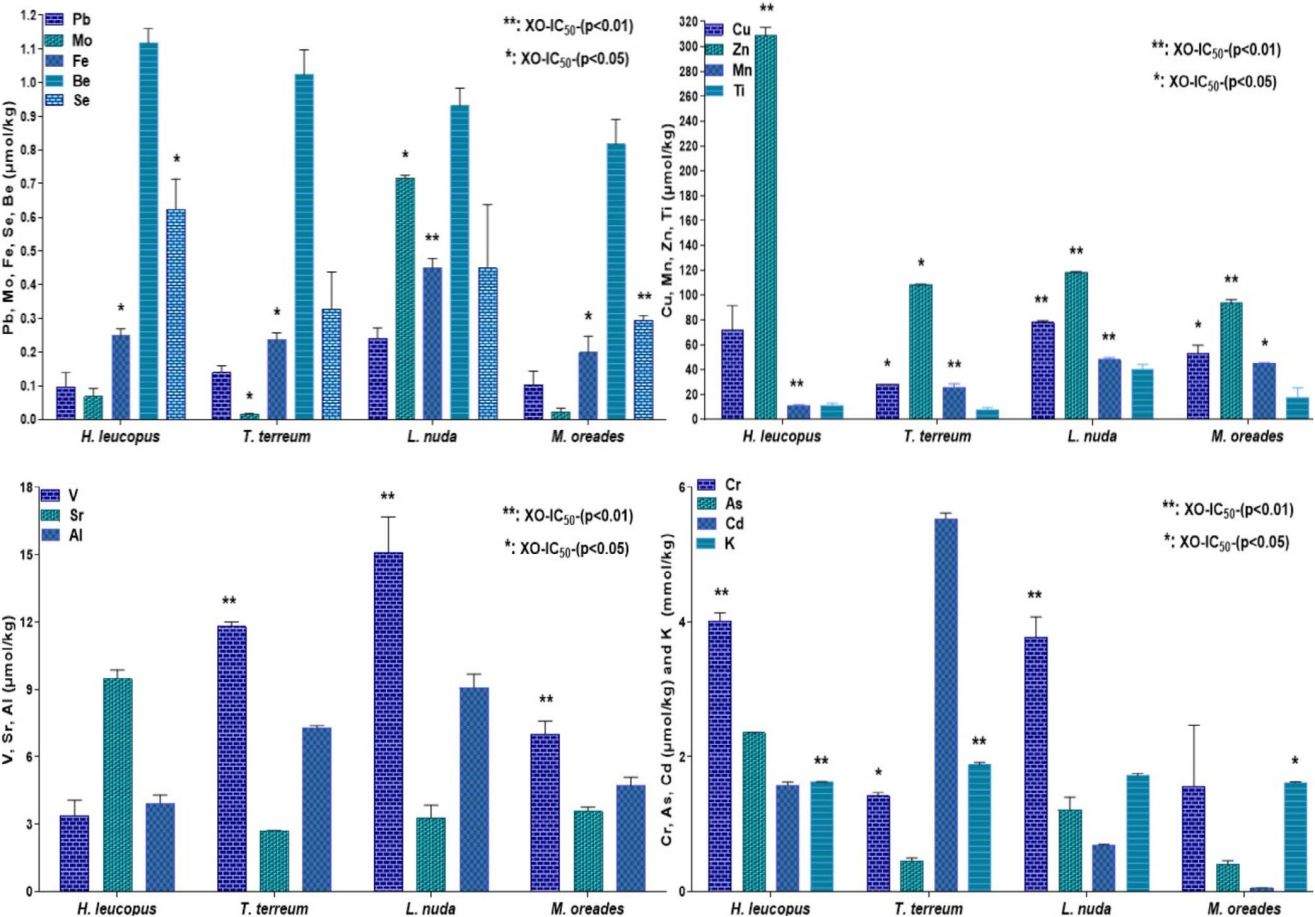
Be, V, Ti, Cr, Cu, Sr., As, Se, Cd, Pb, Mo, Fe, Mn, Al, Zn, K levels. The values are given as mean ± SE of the mean ($\bar{X}$ ± SEM). Significant association between XO inhibitory activity (XO‐IC_50_) and parameters (*p < 0.05, **p < 0.01).

Molybdenum is an essential part of XO. The contents of molybdenum (Mo) are found in four edible mushrooms. *Helvella leucopus, T. terreum, L. nuda*, and *M. oreades* were examined. Regarding Mo, it was found to be slightly higher in *L. nuda* than in other mushrooms. Also, Fe concentrations were found to be slightly higher for *L. nuda* than in other mushroom species (Figure 4). The fact that the inhibitory effect of the enzyme XO could be responsible for the direct scavenging of free radicals requires Mo and Fe as regulators.

XO is a member of the molybdenum protein family and consists of one molybdenum, one flavin adenine dinucleotide, and two ferredoxin‐type iron–sulfur centers (2Fe‐2S) in each of its two independent subunits. The enzyme contains two distinct substrate‐binding sites. XO catalyzes the oxidation of hypoxanthine to xanthine and then to uric acid (Wee et al. 2023). Copper, one of the most important trace elements in the human body, inhibits the activity of XO/xanthine dehydrogenase (XOD/XDH) and thus reduces the oxidation of purine to uric acid. Molybdopterin is the active site of XOD/XDH, which implies that molybdenum is an important component for its activity. In biological terms, there is an antagonistic effect between copper and molybdenum, so increasing copper levels could likely inhibit the activity of XOD/XDH. In addition, XOD/XDH generates a high proportion of ROS with the help of the iron catalyst (Li et al. 2018).

**FIGURE 5:**
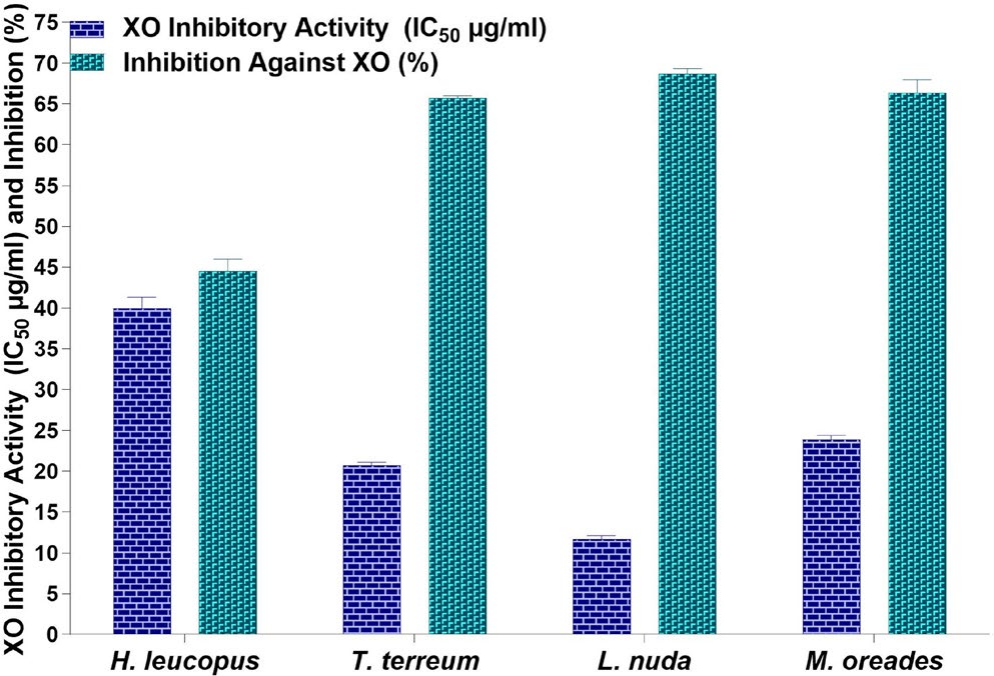
Xanthine oxidase inhibitory activity of four mushroom species (IC_50_ μg/mL) and inhibition against XO (%).

The XO inhibitory activities of four mushrooms of *H. leucopus, T. terreum, L. nuda*, and *M. oreades* were assayed in the methanol extracts. *L. nuda* demonstrated higher inhibitory activity (11.71 ± 0.42 μg/mL) than other mushrooms. *T. terreum, M. oreades*, and *H. leucopus* methanol extracts were determined as 20.71 ± 0.41 μg/mL, 23.85 ± 0.55 μg/mL, and 39.97 ± 1.36 μg/mL, respectively. The highest inhibition percentages were 68.66% ± 0.67% for methanol *L. nuda* extracts and followed by the methanol extract of *H. leucopus, M. oreades*, and *T. terreum* methanol extracts at 44.50% ± 1.50%, 66.33% ± 1.66%, and 65.67% ± 0.33%, respectively (Figure 5). *Lepista nuda* revealed the most potent XO inhibitory activity with an IC_50_ value. This result could be due to the higher total phenolic compounds, phylloquinone, ascorbic acid, total phenolic compounds, and linoleic acid content being responsible for XO inhibitory activities. Thus, the dominant phenolic compound, gallic acid, or other phenolic compounds (hydroxybenzoic acid, ferulic acid, vanillic acid, and trans‐cinnamic acid) primarily provide the in vitro XO inhibitory activities of *L. nuda* extracts. There are no previous values in the literature on the XO inhibitory activity of four edible mushrooms.

The contents of retinol, α‐tocopherol, phylloquinone, and ascorbic acid observed in *H. leucopus, T. terreum, L. nuda*, and *M. oreades* were examined. Regard retinol as much higher in *H. leucopus* than in other mushrooms. The retinol contents of *H. leucopus, T. terreum*, and *L. nuda* ; 0.058 ± 0.003, 0.0473 ± 0.001, and 0.048 ± 0.0007 μmol/kg respectively, regard α‐tocopherol as α‐tocopherol much higher in *T. terreum* than in other mushrooms; the α‐tocopherol concentrations of *H. leucopus, T. terreum, and L. nuda* were 0.6009 ± 0.139, 0.79 ± 0.0064, and 0.3581 ± 0.052 μmol/kg respectively, regard phylloquinone as much higher in *L. nuda* than in other mushrooms; the phylloquinone levels of *T. terreum, L. nuda*, and *M. oreades* were 0.1162 ± 0.025, 0.208 ± 0.0121, and 0.166 ± 0.0058 μmol/kg respectively, regard ascorbic acid as much higher in *L. nuda* than in other mushrooms; the ascorbic acid values were 31.886 ± 3.157, 59.511 ± 7.103, 296.290 ± 48.145, and 7.419 ± 0.789 mg/100 g respectively on a dry weight basis (Table 2). This indicates that *L. nuda* is a more valuable in phylloquinone and ascorbic acid than in the other three mushrooms. Considering the results, it was determined that *T. terreum* had a significant α‐tocopherol content and *H. leucopus* had a high level of retinol. All data are averages of triplicate measurements stated on a dry weight basis. No study on the content of retinol, α‐tocopherol, phylloquinone, and ascorbic acid in *H. leucopus, T. terreum, L. nuda*, and *M. oreades* was found in the literature search. Therefore, we could not compare the data with those from previous literature.

The fatty acid content of the mushroom samples revealed the difference between the species. Palmitic acid, palmitoleic acid, stearic acid, oleic acid, linoleic acid, and eicosadienoic acid were identified in the fruit body of *H. leucopus* samples with proportions of 18.05%, 1.83%, 11.33%, 35.37%, 30.82%, and 2.60%, respectively. In the fruit body of *T. terreum* samples, pentadecylic acid, palmitic acid, palmitoleic acid, stearic acid, oleic acid, linoleic acid, and linolenic acid were found with percentages of 5.57%, 18.78%, 4.19%, 13.51%, 36.31%, 19.92%, and 1.72%, respectively. Pentadecylic acid, palmitic acid, stearic acid, oleic acid, and linoleic acid were observed in the fruit body of *L. nuda* samples with contents of 6.80%, 22.28%, 17.13%, 23.41%, and 30.38%, respectively. In the fruit body of *M. oreades* samples, palmitic acid, palmitoleic acid, stearic acid, oleic acid, and linoleic acid were determined with percentages of 30.30%, 6.31%, 22.92%, 19.61%, and 20.86%, respectively. It has been observed that oleic acid has high levels in *H. leucopus* and *T. terreum* species at 35.37% and 36.31%, respectively. It was linoleic acid in *H. leucopus* species at 30.82%, and palmitic acid in *M. oreades* species with 30.30% as high contents of fatty acids in four mushrooms. Palmitic acid, oleic acid, stearic acid, and linoleic acid are detected in all the species. However, eicosadienoic acid can only be detected in *H. leucopus* with a percentage of 2.60% (Table 3).

The ΣSFA percentage (53.22%) was found higher due to palmitic acid and stearic acid present for *M. oreades* than in other mushroom species. The ΣMUFA proportion (40.50%) was observed higher because of the oleic acid present for *T. terreum* species than in other mushroom species. In addition, the ΣPUFA contents (33.42%) were determined higher because of the linoleic acid present for *H. leucopus* species than in other mushroom species. The proportion of ΣUFAs in *H. leucopus* (70.62%) is higher due to the oleic acid and linoleic acid present than the saturated fatty acids in other mushrooms. Unsaturated fatty acids are essential for human consumption. Some of them (linolenic acid, linoleic acid) are not synthesized in the human body. *Helvella leucopus* and *L. nuda* may be considered a suitable food source due to their highest linoleic acid (omega 6) (30.82%) and (30.38%) contents.

In a study conducted by Turkekul et al., the palmitic acid and linoleic acid percentages of the *T. terreum* are 14.33% and 66.25%. When the *T. terreum* was compared with the *T. terreum* reported by Turkekul et al., it was determined that the palmitic acid content was higher than that of the *T. terreum* mushroom reported by Turkekul. However, the linoleic acid percentage was lower than that of the *T. terreum* mushroom reported by (Turkekul et al. 2017). In a different study, by (Pinto et al. 2013) reported that palmitic acid, stearic acid, and oleic acid contents were low in the *L. nuda* commercial sample at 14.86 ± 0.12, 0.88 ± 0.06, and 16.05 ± 0.40, respectively, compared to this study's values. Furthermore, it is reported that the value of linoleic acid (63.88 ± 0.44) in *L. nuda* is high compared to this study finding.

The structural integrity of all cell membranes depends on linoleic acid and α‐linolenic acid, which are extended and desaturated, in limited quantities, into the longer chain, more polyunsaturated fatty acids that are the precursors to a group of hormone‐like eicosanoid compounds, leukotrienes, and prostaglandins. While α‐linolenic acid (18:3ω‐3) is converted to eicosapentaenoic (20:5ω‐3) and docosahexaenoic (22:6ω‐3) acids, linoleic acid (18:2ω‐6) is converted to arachidonic acid (20:4ω‐6) (Mann and Truswell 2012).

The current study was conducted to evaluate the total antioxidant capacity of *H. leucopus, T. terreum, L. nuda*, and *M. oreades* methanol extracts. The total antioxidant capacity of methanol extracts from *H. leucopus, T. terreum, L. nuda*, and *M. oreades* was observed as 22.150 ± 0.569, 12.755 ± 0.854, 24.712 ± 1.424, and 10.477 ± 1.993 mM ascorbic acid/g, respectively (Table 2). The total phenolic content, indicated as gallic acid equivalents, is related to the antioxidant activity. The total phenolic content was determined in methanol extracts of *H. leucopus, T. terreum, L. nuda*, and *M. oreades* in the present study. The total phenolics of different extracts of mushrooms were determined using Folin–Ciocalteau's assay. The total phenolic content of mushrooms has been documented to have various biological activities, which include antioxidant capacity. The total phenolic contents of the methanol extracts from *H. leucopus, T. terreum, L. nuda*, and *M. oreades* were determined as 2.59 ± 0.079, 3.18 ± 0.451, 2.65 ± 0.027, and 2.76 ± 0.027 mg GA/g, respectively. The total phenolic contents of *T. terreum* were higher than the contents of other mushrooms; these results showed that the higher levels of antioxidant activity were caused by the presence of phenolic components.

Free radicals are reactive molecules that arise spontaneously as by‐products of various daily metabolic processes, such as energy production and immunological reactions (Inci et al. 2023). ROS and free radicals have significant effects on biological cell damage and gene expression. The identified plant antioxidants that protect the cells from free radicals and ROS are essential for the prevention and treatment of cellular oxidation (Selek et al. 2018).

The radical scavenging properties of the methanol extract of *H. leucopus, T. terreum, L. nuda*, and *M. oreades* for anti‐hemolytic activity, superoxide, ABTS, and DPPH radical scavenging activity, which are potent free radicals, were measured, and the antiradical capacities of the mushrooms were tested and compared to trolox and BHT, a synthetic antioxidant.

**FIGURE 6:**
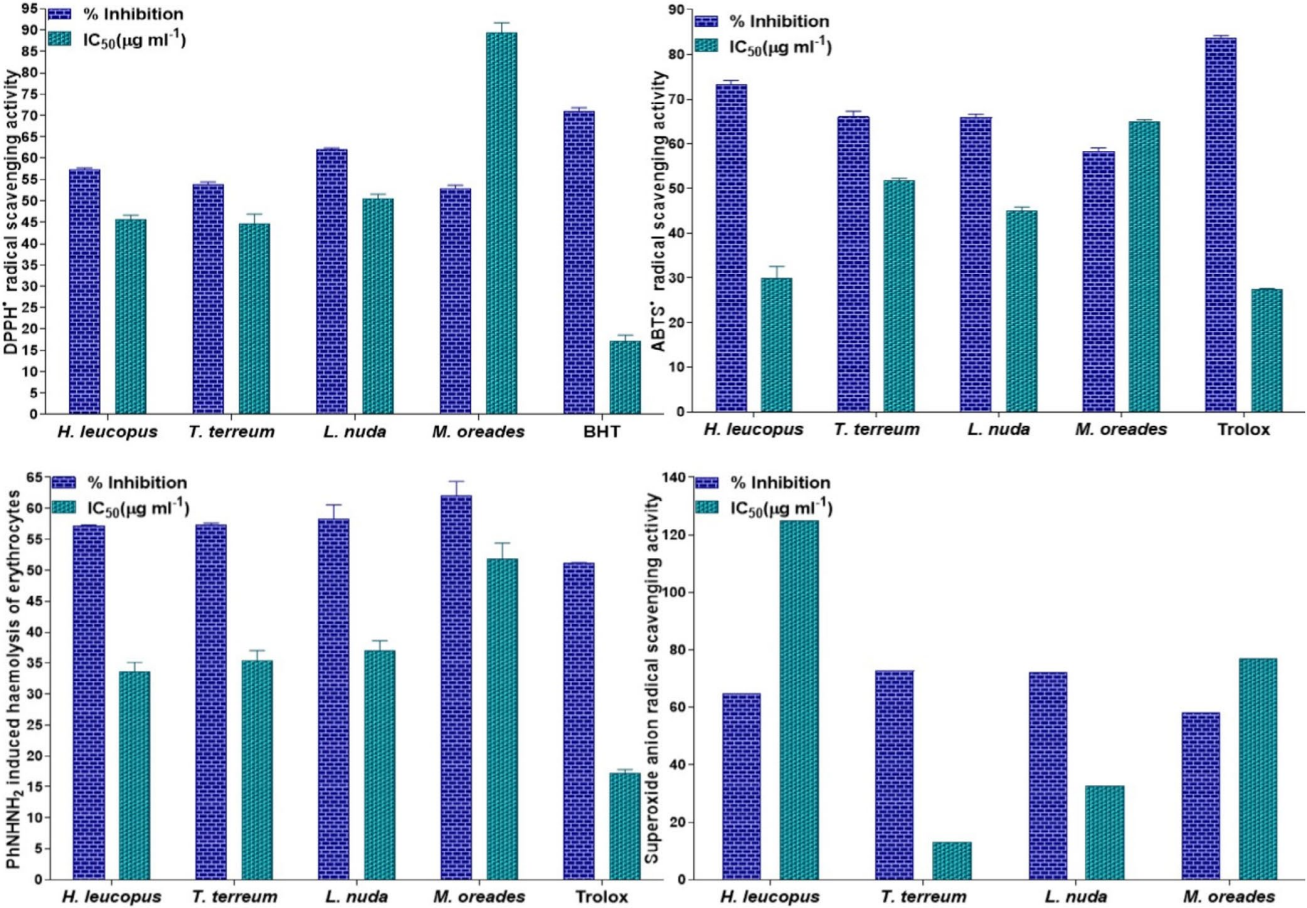
The methanol extract of *Helvella leucopus, Tricholoma terreum, Lepista nuda*, and *Marasmius oreades* of anti‐hemolytic activity, superoxide, ABTS, DPPH radical scavenging activity, trolox and BHT.

The concentration of the sample required to decrease the starting concentration of DPPH by 50% (IC_50_) within the experimental situations was measured. A lower level of IC_50_ shows higher antioxidant activity. The highest free radical scavenging activity was observed with the *T. terreum* methanolic extract (IC_50_ 44.619 ± 2.315 μg/mL), while the *H. leucopus, L. nuda*, and *M. oreades* methanol extracts indicated comparable levels of free radical scavenging activity with IC_50_ values of 45.562 ± 1.126, 50.535 ± 1.029, and 89.321 ± 2.37 μg/mL. It was seen in our study that DPPH scavenging activity (%), *H. leucopus, T. terreum, L. nuda*, and *M. oreades* were determined at 57.531% ± 0.247%, 53.981% ± 0.401%, 62.037% ± 0.370%, and 53.055% ± 0.586% respectively. BHT showed comparable free radical scavenging activity with an IC_50_ value of 17.064 ± 1.517 μg/mL, and a percentage Inhibition of 71.11% ± 0.694%. It can be assumed that the methanolic extract of the *T. terreum* showed, as DPPH, more potent in vitro antioxidant activity than the other mushrooms methanol extracts (Table 4, Figure 6).

The ABTS radical scavenging activity was found with *H. leucopus* methanol extract (IC_50_ 30.011 ± 2.548 μg/mL), while the *T. terreum, L. nuda*, and *M. oreades* methanol extracts were determined as 51.622 ± 0.745, 44.892 ± 1.029, and 65.062 ± 0.365 μg/mL. The highest ABTS radical inhibition rates were 73.397% ± 0.801% for *H. leucopus* methanol extracts and 66.031% ± 1.336%, 65.935% ± 0.668%, and 58.333% ± 0.801% for *T. terreum, L. nuda*, and *M. oreades* methanol extracts. Trolox was determined as (IC_50_ 27.464 ± 0.212 μg/mL) and a percentage inhibition of 83.696% ± 0.544%. *Helvella leucopus* methanol extract was more effective in scavenging radicals than other mushroom methanol extracts.

Superoxide anion scavenging activities were obtained with *T. terreum* methanol extract (IC_50_ 12.89234 μg/mL), while the *H. leucopus, L. nuda*, and *M. oreades* methanol extracts were determined as 124.977, 32.70621, and 77.04739 μg/mL, respectively. Superoxide anion scavenging activities of the *H. leucopus, T. terreum, L. nuda*, and *M. oreades* methanol extracts, *H. leucopus, T. terreum, L. nuda*, and *M. oreades* accounted for inhibition of 65%, 73%, 72%, and 58%, respectively. The positive control was not tested. It can be considered that the methanol extract of *T. terreum* exhibits an antioxidant effect, which was demonstrated by the scavenging of superoxide radicals. In the current study, potent antioxidant activity was observed in the methanolic extract of *T. terreum*, which revealed superoxide radical scavenging activity using the xanthine‐XO system with an IC_50_ of 12.89234 μg/mL.

The PhNHNH_2_‐induced haemolysis of erythrocytes radical scavenging activity was observed with *H. leucopus* methanol extract (IC_50_ 33.68 ± 1.43 μg/mL), while the *T. terreum, L. nuda*, and *M. oreades* methanol extracts were determined as 35.34 ± 1.75, 37.09 ± 1.54, and 51.88 ± 2.53 μg/mL, respectively. The highest PhNHNH2‐induced hemolysis rates were 62.08% ± 2.28% for *M. oreades* methanol extracts and 57.18% ± 0.15%, 57.36% ± 0.25%, and 58.36% ± 2.23% for *H. leucopus, T. terreum*, and *L. nuda* methanol extracts. Trolox was used as the positive control. Trolox was determined as (IC_50_ 17.24 ± 0.54 μg/mL) and a percentage Inhibition of 51.14% ± 0.10%. *H. leucopus* methanol extract was more effective in anti‐hemolytic activity than other mushroom methanol extracts. Current results of total antioxidant capacity indicate that methanol extracts of *M. oreades* were lower than those of other mushrooms. *T. terreum m*ethanol extract showed a high total antioxidant activity. *H. leucopus* has less total antioxidant activity than *M. oreades m*ethanol extract.

*Lepista nuda* is documented as a rich source of proteins as well as several bioactive compounds, such as polysaccharides, vitamins, essential amino acids, carbohydrates, and riboflavin, which are beneficial for human consumption (Deshmukh et al. 2023). *Lepista nuda* demonstrated higher inhibitory activity than other mushrooms. *L. nuda* revealed potent XO inhibitory activity with IC_50_ value. *Lepista nuda* has total phenolic compounds, phylloquinone, ascorbic acid, total phenolic compounds, and linoleic acid content that are responsible for high XO inhibitory activities.

A correlation test was used to analyze the relationships found in the edible mushroom species (*H. leucopus, T. terreum, L. nuda, and M. oreades*) between XO inhibitory activity (XO‐IC_50_) and significant associated contents of gallic acid, *o*‐coumaric acid, hydroxybenzoic acid, vanillic acid, ferulic acid, trans‐cinnamic acid, resveratrol, retinol, ascorbic acid, phylloquinone, α‐tocopherol, ascorbic acid, Cr, Se, Fe, Mn, Zn, K, V, Cu, Mo.

The results of the analysis of four mushrooms, positive strong correlations were observed among XO‐IC_50_ with gallic acid, *o*‐coumaric acid, retinol, ascorbic acid, Cr, Se, Fe, Mn, Zn, K in the *H. leucopus* (*r* = 0.953; *p* < 0.05), (*r* = 0.972; *p* < 0.05), (*r* = 0.993; *p* < 0.01), (*r* = 0.983; *p* < 0.05), (*r* = 0.996; *p* < 0.01), (*r* = 0.969; *p* < 0.05), (*r* = 0,986; *p* < 0.05), (*r* = 0.996; *p* < 0.01), (*r* = 0.997; *p* < 0.01), (*r* = 0.999; *p* = 0.001). Positive significant strong associations were found among XO‐IC_50_ with gallic acid, hydroxybenzoic acid, *o*‐coumaric acid, resveratrol, α‐tocopherol, retinol, ascorbic acid, V, Cr, Cu, Mo, Fe, Mn, Zn, and K in the *T. terreum* (*r* = 0.999; *p* = 0.01), (*r* = 0.995; *p* < 0.01), (*r* = 0.999; *p* = 0.001), (*r* = 0.998; *p* < 0.01), (*r* = 1; *p* < 0.001), (*r* = 0.997; *p* < 0.01), (*r* = 0.990; *p* = 0.01), (*r* = 0.996; *p* < 0.01), (*r* = 0.998; *p* < 0.05), (*r* = 0.999; *p* < 0.05), (*r* = 0.988; *p* < 0.05), (*r* = 0.989; *p* < 0.05), (*r* = 0.994; *p* < 0.01), (*r* = 0.999; *p* < 0.05), and (*r* = 0.998; *p* < 0.01). Moreover, strong positive associations were observed among XO‐IC_50_ with gallic acid, hydroxybenzoic acid, vanillic acid, ferulic acid, *o*‐coumaric acid, trans‐cinnamic acid, phylloquinone, α‐tocopherol, retinol, ascorbic acid, V, Cr, Cu, Mo, Fe, Mn, and Zn in the *L. nuda* (*r* = 1; *p* < 0.001), (*r* = 0.979; *p* < 0.05), (*r* = 0.943; *p* < 0.05), (*r* = 0.989; *p* = 0.005), (*r* = 0.993; *p* < 0.01), (*r* = 0.972; *p* < 0.05), (*r* = 0.999; *p* < 0.05), (*r* = 0.968; *p* < 0.05), (*r* = 0.999; *p* < 0.05) (*r* = 0.985; *p* < 0.01), (*r* = 0.980; *p* = 0.01), (*r* = 0.987; *p* < 0.01), (*r* = 0.997; *p* < 0.01), (*r* = 0.999; *p* < 0.05), (*r* = 0.990; *p* < 0.01), (*r* = 0.996; *p* < 0.01), (*r* = 0.999; *p* = 0.001). In the present study, significant strong positive correlations were also found among XO‐IC_50_ with trans‐cinnamic acid, phylloquinone, ascorbic acid, V, Cu, Se, Fe, Mn, Zn, and K in the *M. oreades* (*r* = 0.975; *p* < 0.05), (*r* = 0.997; *p* < 0.01), (*r* = 0.993; *p* < 0.01), (*r* = 0.997; *p* < 0.01), (*r* = 0.981; *p* < 0.05), (*r* = 0.996; *p* < 0.01), (*r* = 0.959; *p* < 0.05), (*r* = 0.998; *p* < 0.05), (*r* = 0.997; *p* < 0.01), and (*r* = 0.999; *p* < 0.05).

These results indicate that XO inhibitory activity is related to the levels of Mo and Fe and plays a significant role in the activity of the XO enzyme in the *T. terreum and L. nuda*. Therefore, Mo and Fe clearly show the contributions and relationships of XO inhibitory activity. In addition, the correlation test for *T. terreum and L. nuda* revealed that the XO inhibitory activity with among gallic acid, *o*‐coumaric acid, hydroxybenzoic acid, vanillic acid, ferulic acid, trans‐cinnamic acid, resveratrol, retinol, α‐tocopherol, ascorbic acid, Se, and Zn with antioxidant properties, it is seen that they important play a protective role both in relation to XO inhibitory activity and against other diseases related to this enzyme.

Edible four mushrooms have medicinal properties such as inhibitory activities against XO, antioxidant capacity, and antihaemolytic activities, due to rich antioxidant compounds such as phenolic compounds, unsaturated fatty acids, some vitamins (retinol, and α‐tocopherol) and trace elements (Se and Zn), which are able to prevent negative effects of oxidative stress.

The results of the measurements showed that the four mushrooms had very high K content and high amounts of Fe, Zn, Cu, and Mn. *Lepista nuda* is a better source of phylloquinone and ascorbic acid than the other three mushrooms. *Lepista nuda* also contains the total phenolic compounds at the highest level. The total antioxidant, phenolic, and α‐tocopherol contents of *T. terreum* were higher than the contents of other mushrooms. It is also a very effective in DPPH and superoxide radical scavenging activity. *Helvella leucopus* has a high level of retinol. *Helvella leucopus* also, methanol extract was more effective in anti‐hemolytic activity than other mushrooms. It is quite strong in ABTS radical scavenging activity. *Helvella leucopus* and *L. nuda* may be considered a suitable food sources due to their highest linoleic acid contents. *Lepista nuda* contains the analyzed total phenolic compounds at the highest level and this is followed by *T. terreum*, *M. oreades* , and *H. leucopus*. The phenolic compounds and vitamins (α‐tocopherol, and retinol) seem to be the primary components responsible for the antioxidant effect of the extracts from the four mushroom species. The four mushroom species can be used as a potential supplement or in the pharmaceutical and cosmetic industries as a source of natural antioxidants.

The fruiting bodies of four edible mushrooms have values of gallic acid, *o*‐coumaric acid, and trans‐cinnamic acid at a significantly high level. Only a small quantity of vanillic acid was determined. In this study, it was found that the phytochemical constituents of methanol extracts in the four mushrooms have found the existence of gallic acid, *o*‐coumaric acid, and trans‐cinnamic acid linked to their anti‐XO activity and antioxidant capacity. Unsaturated fatty acids, vitamins, some trace elements, and phenolic compounds are very important for the efficient functions of the body.

## 5 Conclusions

Edible two medicinal mushrooms ( *L. nuda* and *T. terreum*) have medicinal properties such as XO inhibitory activities, antioxidant capacity, and antihaemolytic activities, due to rich antioxidant compounds such as phenolic compounds, some vitamins, and trace elements, which could be a suitable prevention against negative effects of oxidative stress. The present study shows a correlation between XO inhibitory activity and gallic acid, *o*‐coumaric acid, hydroxybenzoic acid, vanillic acid, ferulic acid, trans‐cinnamic acid, resveratrol, retinol, α‐tocopherol, ascorbic acid, Se, and Zn in the *T. terreum and L. nuda*. In this study, it was also found that unsaturated fatty acids might play an important role in the inhibitory activity of the XO enzyme in *T. terreum and L. nuda*. The fact that the inhibitory effect of the enzyme XO could be linked to the direct scavenging of free radicals requires Mo and Fe as regulators. The levels of retinol, α‐tocopherol, Se, Zn, gallic acid, *o*‐coumaric acid, hydroxybenzoic acid, vanillic acid, ferulic acid, trans‐cinnamic acid, and resveratrol in *L. nuda* and *T. terreum* could potentially be responsible for preventing free radical damage due to their antioxidant properties in relation to XO inhibitory activity for hyperuricemia, gout, as well as against other diseases XO‐related diseases induced by ROS.

## Author Contributions

**Suat Ekin:** conceptualization (equal), formal analysis (lead), investigation (lead), resources (equal), writing – original draft (lead), writing – review and editing (lead). **Mahire Bayramoglu Akkoyun:** investigation (equal), methodology (equal), resources (equal). **Ahmet Bakir:** investigation (supporting), methodology (supporting). **Mustafa Emre Akcay:** formal analysis (supporting), investigation (supporting). **Emre Can Ekin:** investigation (equal), methodology (equal).

## Conflicts of Interest

The authors declare no conflicts of interest.

## Supporting information


**Figure S1.** HPLC chromatogram of retinol, and α‐tocopherol for *H. leucopus*.
**Figure S2.** HPLC chromatogram of retinol, α‐tocopherol, and phylloquinone for *T. terreum*.
**Figure S3.** HPLC chromatogram of retinol, α‐tocopherol, and phylloquinone for *L. nuda* .
**Figure S4.** HPLC chromatogram of phylloquinone for *M. oreades* .
**Figure S5.** The gallic acid, protocatechuic acid, hydroxybenzoic acid, vanillic acid, o‐coumaric acid, resveratrol, trans‐cinnamic acid chromatograms for *H. leucopus*.
**Figure S6.** The gallic acid, hydroxybenzoic acid, vanillic acid, o‐coumaric acid, resveratrol chromatograms for *T. terreum*.
**Figure S7.** The gallic acid, hydroxybenzoic acid, vanillic acid, Feulic acid, o‐coumaric acid, trans‐cinnamic acid chromatograms for *L. nuda* .
**Figure S8.** The gallic acid, o‐coumaric acid, trans‐cinnamic acid chromatograms for *M. oreades*.

## Data Availability

The data that support the findings of this study are available on request from the corresponding author.
