# Supplementary material for: Comparison of Xanthine Oxidase Inhibitory Activities and Phenolic, Fatty Acid, Element, and Vitamin Levels of Four Mushroom Species
Source: Food Sci Nutr. 2025 Apr 21;13(4):e70203. doi: 10.1002/fsn3.70203 (PMC12011987; doi:10.1002/fsn3.70203)
Supplement: Supplementary file 1 — Figure S1. HPLC chromatogram of retinol, and α‐tocopherol for H. leucopus. Figure S2. HPLC chromatogram of retinol, α‐tocopherol, and phylloquinone for T. terreum. Figure S3. HPLC chromatogram of retinol, α‐tocopherol, and phylloquinone for L. nuda . Figure S4. HPLC chromatogram of phylloquinone for M. oreades . Figure S5. The gallic acid, protocatechuic acid, hydroxybenzoic acid, vanillic acid, o‐coumaric acid, resveratrol, trans‐cinnamic acid chromatograms for H. leucopus. Figure S6. The gallic acid, hydroxybenzoic acid, vanillic acid, o‐coumaric acid, resveratrol chromatograms for T. terreum. Figure S7. The gallic acid, hydroxybenzoic acid, vanillic acid, Feulic acid, o‐coumaric acid, trans‐cinnamic acid chromatograms for L. nuda . Figure S8. The gallic acid, o‐coumaric acid, trans‐cinnamic acid chromatograms for M. oreades. [file FSN3-13-e70203-s001.docx]

**Supplementary materials**

**Xanthine oxidase inhibitory activities, phenolic compound, fatty acid, trace element, and vitamin contents of four edible mushroom species**

Suat Ekin^1*^, Mahire Bayramoglu Akkoyun^2^, Ahmet Bakir^1^, Mustafa Emre Akcay^3^,

Emre Can Ekin^4^

^1*^Department of Chemistry, Science Faculty, Van Yuzuncu Yil University, Van, Turkiye

^2^Division of Biochemistry, Faculty of Veterinary Medicine, Siirt University, Siirt, Turkiye

^3^Yuzuncu Yil University, Faculty of Science, Department Biology, Van, Turkiye

^4^Nursing Department, Faculty of Health Sciences, Istanbul Arel University, Istanbul, Turkiye


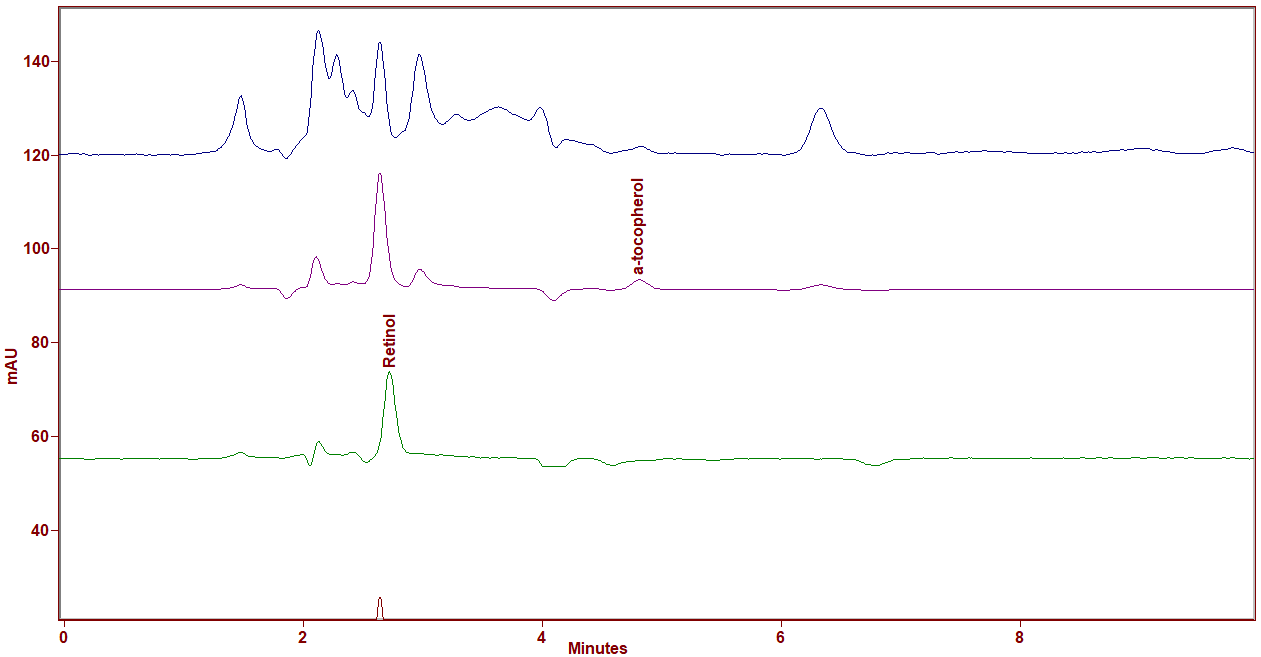


**SUPPLEMENTARY FIGURE S1** HPLC chromatogram of retinol, and α-tocopherol for *H. leucopus.*


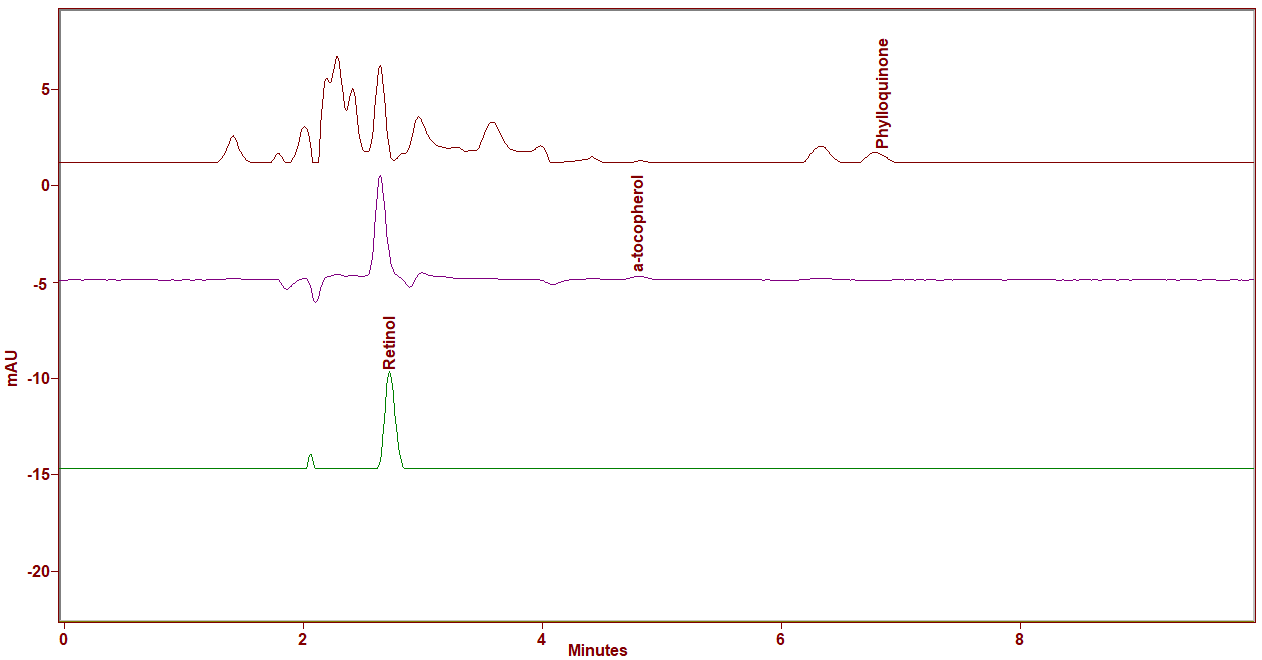


**SUPPLEMENTARY FIGURE S2** HPLC chromatogram of retinol, α-tocopherol, and phylloquinone for *T. terreum*.


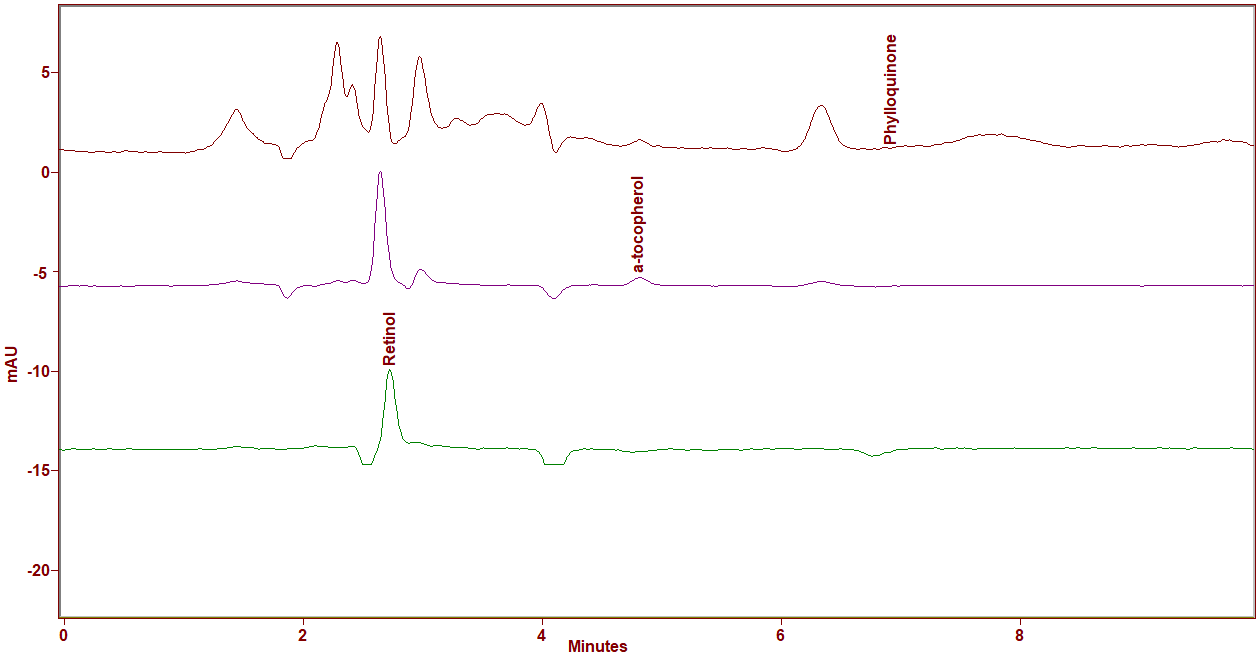


**SUPPLEMENTARY FIGURE S3** HPLC chromatogram of retinol, α-tocopherol, and phylloquinone for *L. nuda*.


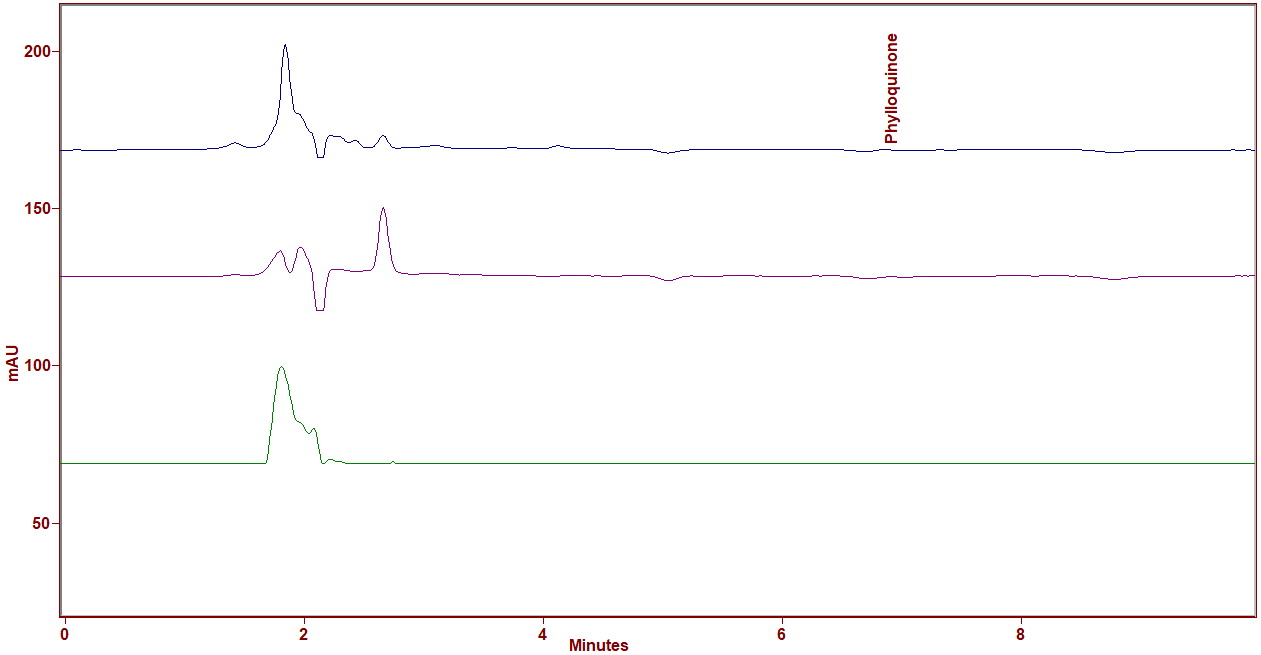


**SUPPLEMENTARY FIGURE S4** HPLC chromatogram of phylloquinone for *M. oreades*.


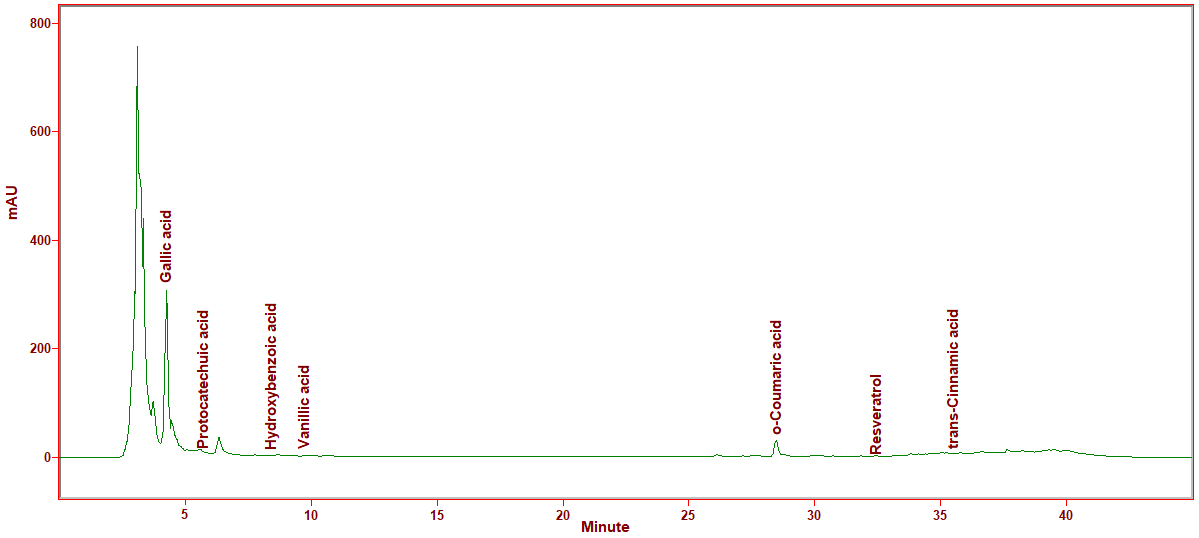


**SUPPLEMENTARY FIGURE S5** The gallic acid, protocatechuic acid, hydroxybenzoic acid, vanillic acid, o-coumaric acid, resveratrol, trans-cinnamic acid chromatograms for *H. leucopus.*


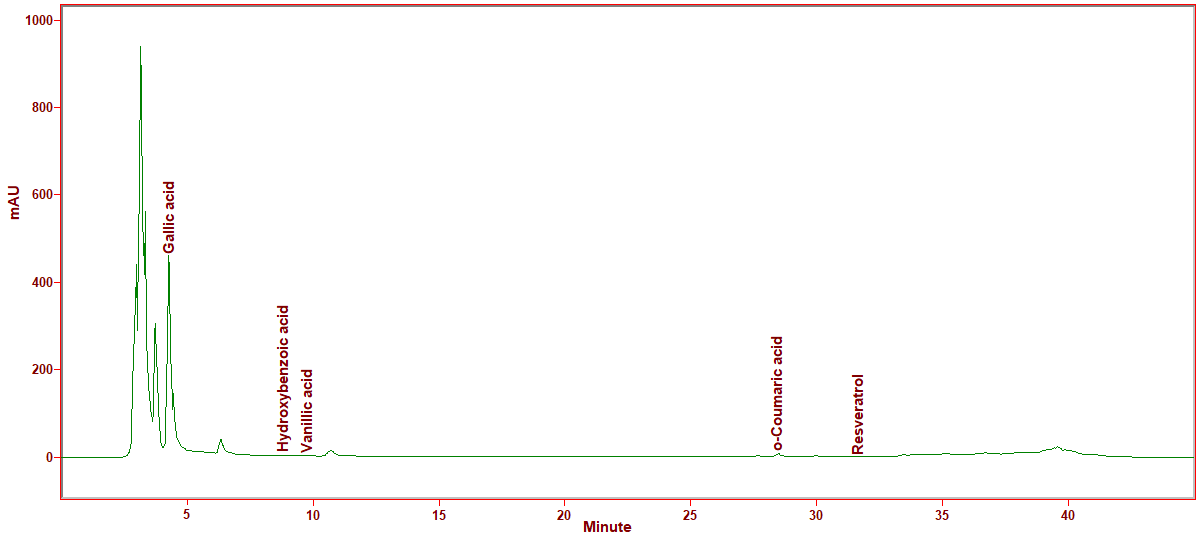


**SUPPLEMENTARY FIGURE S6** The gallic acid, hydroxybenzoic acid, vanillic acid, o-coumaric acid, resveratrol chromatograms for *T. terreum*.


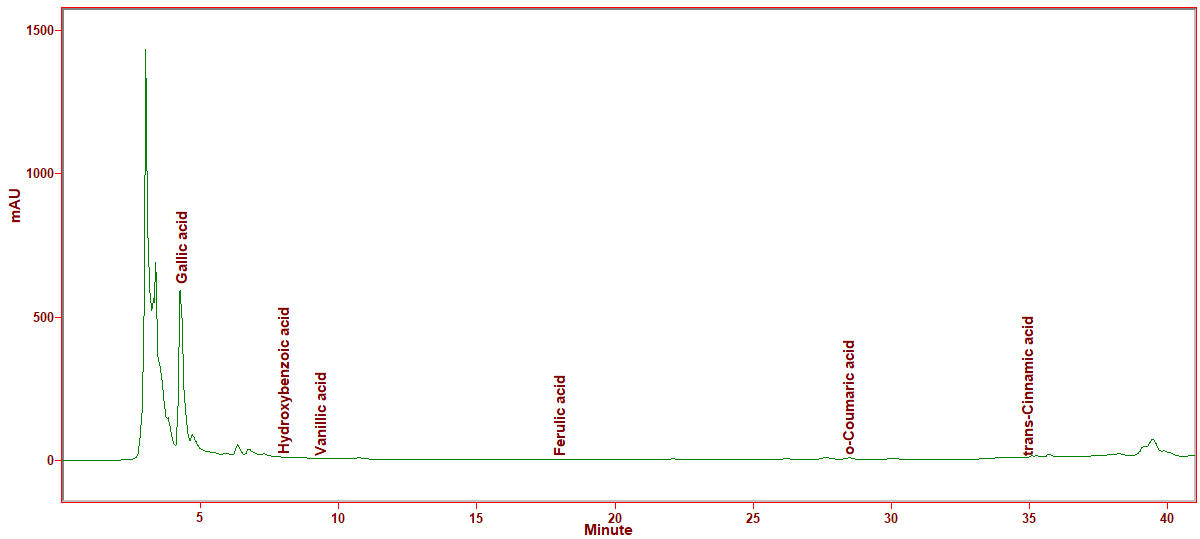


**SUPPLEMENTARY FIGURE S7** The gallic acid, hydroxybenzoic acid, vanillic acid, Feulic acid, o-coumaric acid, trans-cinnamic acid chromatograms for *L. nuda*.


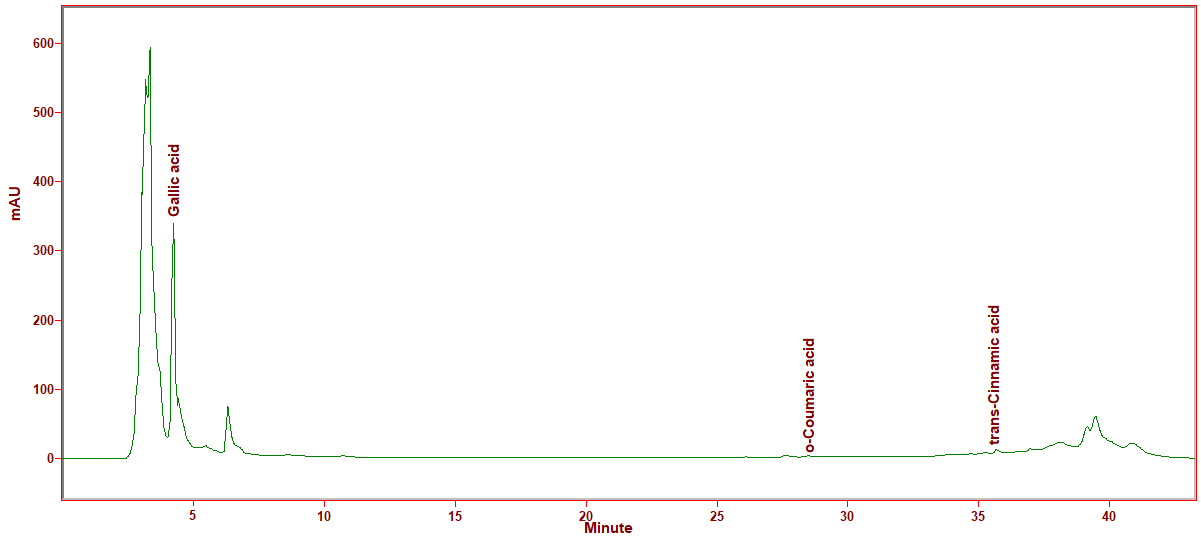


**SUPPLEMENTARY FIGURE S8** The gallic acid, o-coumaric acid, trans-cinnamic acid chromatograms for *M. oreades.*
